# ER stress induced extracellular vesicles secretion from macrophages promotes calcium oxalate crystals formation in kidney

**DOI:** 10.1186/s43556-025-00351-x

**Published:** 2025-11-17

**Authors:** Yiqiong Yuan, Yucheng Ma, Lunzhi Dai, Xi Jin, Shiqian Qi, Zhaofa Yin

**Affiliations:** 1 Department of Urology, Institute of Urology (Laboratory of Reconstructive Urology), West China Hospital, Sichuan University, No. 37 Guo Xue Xiang, Chengdu, Sichuan 610041 P.R. China; 2 State Key Laboratory of Biotherapy, West China Hospital, Sichuan University, and National Collaborative Innovation Center, Chengdu, Sichuan P.R. China; 3 Department of Urology, Loudi Central Hospital of Hunan Province, No. 51 Changqing East Street, Loudi, Hunan 417000 P.R. China

**Keywords:** Endoplasmic reticulum stress, Macrophages, Kidney stones, Extracellular vesicles, Lysosome function

## Abstract

Macrophages are involved in the development of kidney stone disease, and substantial research suggests that macrophage-derived extracellulr vesicles (Mφ-EVs) may play a pivotal role in this process. However, the impact of Mφ-EVs on kidney stone formation and the mechanisms of EVs secretion remain unclear. Here we found that calcium oxalate (CaOx) crystals stimulation leads to increased inflammation and enhanced secretion of extracellular vesicles (EVs) in macrophages. Interestingly, these EVs can be absorbed by renal cells, significantly promoting renal tubular damage and facilitating the formation of CaOx crystals in mouse kidneys. Mechanistically, CaOx crystal stimulation activates endoplasmic reticulum stress (ERS)-related proteins, particularly PKR-like ER kinase (PERK), resulting in the production of EVs containing glucose-regulated protein 94 (GRP94). Concurrently, this process induces lysosomal dysfunction, inhibiting the degradation of EVs by lysosomes and ultimately leading to their release. Knocking down PERK in macrophages could reduce the Mφ-EVs secretion significantly, which decreased in the accumulation of CaOx crystals in the mouse kidneys. Together, our research uncovers a novel mechanism by which CaOx crystal-induced ERS and lysosomal dysfunction in macrophages promote the secretion of EVs, thereby facilitating kidney stone formation. This finding presents a promising therapeutic target for future interventions.

**Supplementary Information:**

The online version contains supplementary material available at 10.1186/s43556-025-00351-x.

## Introduction

The prevalence of kidney stone disease (KSD) in the United States is reported to be 11% [1], while in China, it is reported to be 11.4% [1, 2]. Calcium oxalate stones account for about 80% of cases, with a recurrence rate of approximately 60% within ten years [2, 3]. However, the pathogenesis of KSD remains unclear. Hence, investigating the mechanisms underlying kidney stone formation and developing new therapies are crucial for preventing the recurrence of KSD.

Excessive CaOx crystals can lead to tubular injury and intrarenal inflammation, resulting in crystal deposition, nephrocalcinosis, and the development of urolithiasis [4, 5]. Tubular damage may impair the reabsorption of substances such as calcium and uric acid, leading to increased levels in urine and promoting stone formation [6]. Renal tubular injury play a crucial role in the formation of kidney stones. Cell debris or substances resulting from the degradation of injured renal tubular epithelial cells can serve as substrates for heterogeneous nucleation, promoting crystal formation [7, 8]. Additionally, damaged renal tubular epithelial cells with intact cell membrane structures can also enhance the nucleation of calcium oxalate stones [9]. Recent studies have identified a type of cell with high expression of injury markers in the renal papillae of kidney stone patients [10]. Similarly, other research has demonstrated significant expression of damage-related proteins and cell adhesion proteins, such as Clusterin, HSP27, and Collagen, in the matrix of calcium oxalate stones [11, 12]. Thus, renal tubular injury not only creates conditions conducive to the heterogeneous nucleation of calcium oxalate stones but also provides sites for the adhesion of calcium oxalate crystals, facilitating stone formation.

Macrophages are crucial immune cells distributed throughout the body, capable of engulfing and digesting harmful materials [13–19]. The activities of macrophages are regulated by stimulation from harmful materials, such as CaOx crystals [20, 21]. A recent study demonstrated that macrophages phagocytose precipitated particles in the renal medulla to alleviate inflammation [22]. However, in kidney diseases, macrophages can exacerbate tissue inflammation through autocrine, paracrine, and cytokine mechanisms [23, 24]. Notably, paracrine signaling has been implicated in various functions of extracellular vesicles (EVs) [25, 26]. Macrophage-derived extracellular vesicles (Mφ-EVs) have emerged as important mediators in the pathology of multiple diseases, including inflammatory conditions, fibrosis, and cancers [27]. The composition of EVs from CaOx kidney stone patients differs significantly from that of healthy controls [28]. These findings underscore the importance of macrophages and EV secretion in kidney stone formation [29–31]. However, the functional changes in macrophages after they are stimulated by CaOx crystals and the specific mechanism of Mφ-EVs secretion in KSD are still unclear.

This study aims to elucidate the impact of Mφ-EVs on stone formation and the mechanisms of EVs secretion. We hypothesize that CaOx crystal promotes EVs secretion, which exacerbates renal tubular injury and inflammation, thereby facilitating stone formation. We found that the phagocytosis of CaOx crystals by macrophages leads to the secretion of Mφ-EVs, which in turn promotes CaOx crystal formation in mouse kidneys. Furthermore, we revealed that the protein kinase RNA-like endoplasmic reticulum kinase (PERK, also known as *Eif2ak3*) triggered lysosomal dysfunction in macrophages, which initiated the release of EVs. Knocking down PERK in macrophages alleviated kidney stone formation in mouse models. These findings revealed the role of ER stress in Mφ-EVs release, providing insights into the mechanisms underlying KSD and potential prevention strategies. This research provides insights into the mechanisms underlying KSD and potential prevention strategies. This research contributes to understanding the pathophysiology of kidney stones and offers new avenues for intervention.

## Results

### The phagocytosis of COM by macrophages leads to a proinflammatory phenotype

To explore the phenotypic changes in macrophages induced by calcium oxalate (CaOx) in nephrolithiasis using an in vitro model, we first compared the alterations in macrophages in mice induced by CaOx with those in BMDMs (marrow-derived macrophages) stimulated by COM (calcium oxalate monohydrate), a uniform CaOx crystal prepared in vitro. We synthesized CaOx crystals, which were identified via infrared spectroscopy (Fig. S1a). The penetrating twin CaOx crystals, measuring 3–6 µm, are named COM (Fig. S1b). BMDMs were isolated, and their morphology and F4/80 expression were characterized on days 3 and 6 using optical microscopy and flow cytometry (FCM) (Fig. S1c-d). Furthermore, FCM was used to detect the changes in TLR4 and iNOS in renal macrophages from mice at days 0, 3, and 7 following administered glyoxylic acid (GLA) modeling, as well as in COM-stimulated BMDM cells after 24 h. The results revealed a time-dependent upregulation of both TLR4 and iNOS expression in renal macrophages throughout crystal deposition progression (Fig. S1e-f). The polarized phenotype of BMDMs exhibited comparable changes in vivo, primarily reflected in the positive rates of iNOS and TLR4 markers (Fig. S1g-h).

**Fig. 1:**
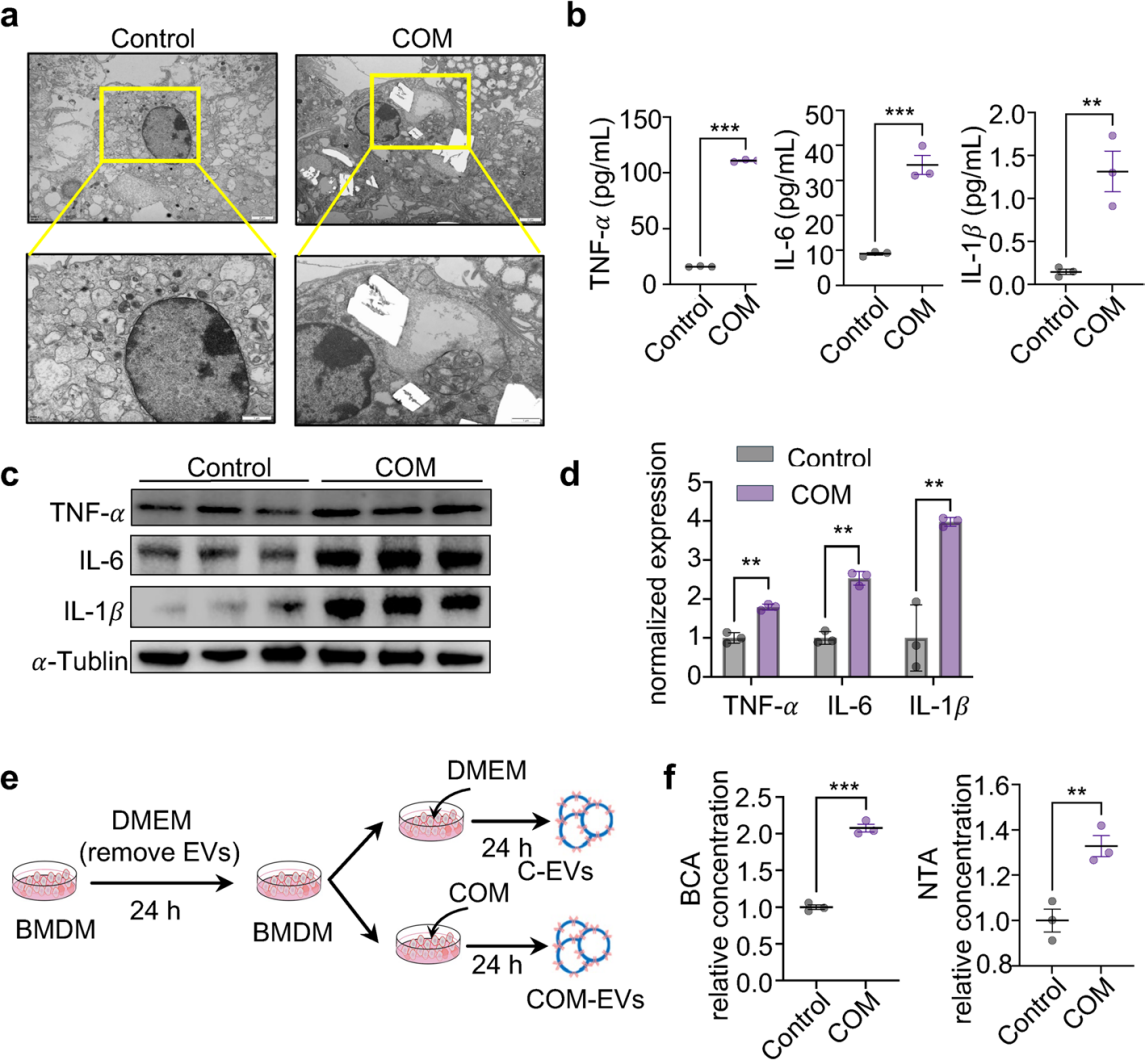
The phagocytosis of COM by macrophages leads to inflammation. **a** Transmission electron microscopy (TEM) images of BMDMs treated with or without COM for 24 h. **b** ELISA analysis of the cytokine levels of TNF-α, IL-6 and IL-1β in the supernatants of BMDMs treated with or without COM for 24 h. **c**-**d** Western blot analysis of the protein levels of TNF-α, IL-6 and IL-1β in BMDMs treated with or without COM for 24 h. Relative quantification (**d**) of the data presented in (**c**). **e** Schematic diagram of how EVs were isolated from the supernatant of BMDMs with or without COM. **f** The concentrations of EVs released by BMDMs with or without COM treatment for 24 h were evaluated by BCA (left) or NTA (right). EVs particle and protein concentrations were normalized to the total protein content of cell. Data from the control group were set to 1, and experimental groups are expressed as fold changes relative to the control. All data are presented as means ± SEM. Significance was determined by unpaired Student’s t test, *n* = 3 biologically independent samples, ***p < 0.001, **p < 0.01, *p < 0.05

Subsequently, to investigate the phenotypic alterations of macrophages during COM stimulation, we examined the internal morphology, inflammation, and vesicles secretion of BMDMs. Transmission electron microscopy (TEM) analysis confirmed that BMDMs could phagocytose COM (Fig. 1a). Additionally, the levels of inflammatory factors, including TNF-α (tumor necrosis factor alpha), IL-6 (interleukin 6) and IL-1β (interleukin-1beta), significantly increased following COM stimulation (Fig. 1b). Furthermore, the protein levels of TNF-α, IL-6 and IL-1β in BMDMs were markedly elevated after COM stimulation (Fig. 1c-d). We obtained extracellular vesicles (EVs) from the supernatant of BMDMs through ultracentrifugation. The EVs secreted by the untreated medium group were designated as C-EVs, while those from the COM stimulation group were termed COM-EVs (Fig. 1e). Bicinchoninic acid (BCA) and nanoparticle tracking analyzer (NTA) analyses revealed a significant increase in EVs derived from macrophages with COM stimulation (Fig. 1f). Collectively, these data suggest that the phagocytosis of COM by macrophages induces a proinflammatory phenotype and results in the release of EVs.

### EVs from BMDMs with COM stimulation promote renal tubular epithelium injury

**Fig. 2:**
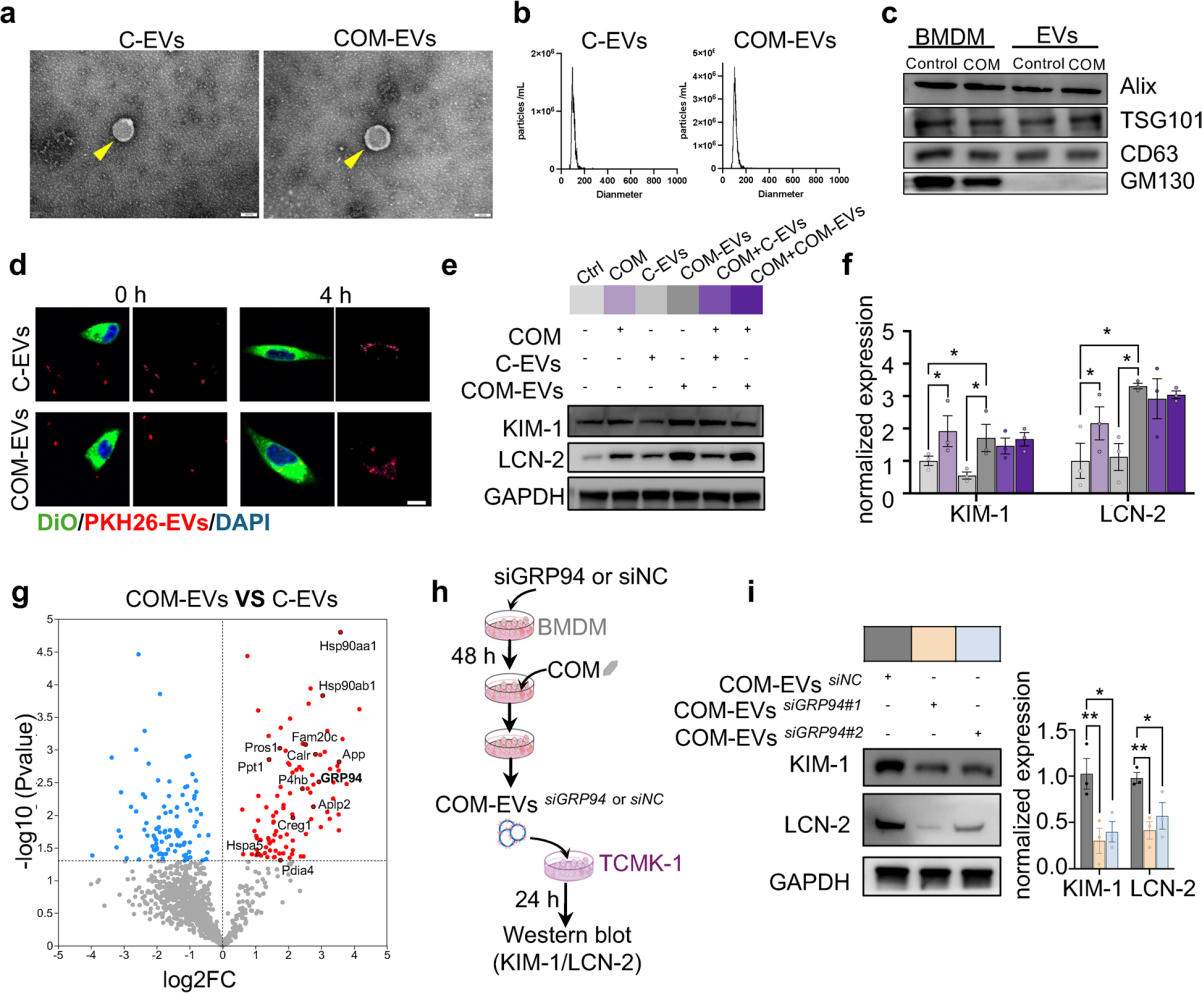
EVs from BMDMs with COM stimulation promote tubular epithelium injury. **a**-**c** EVs were isolated and characterized from the supernatant of BMDMs treated with or without COM for 24 h. **a** TEM images of EVs. **b** NTA analysis of the size distributions of EVs. **c** Western blot analysis of the EVs markers (ALIX, TSG101 and CD63) and the Golgi marker (GM130). **d**-**f** BMDMs derived EVs were incubated with TCMK-1 cells. **d** Representative fluorescence images of PKH26-labeled EVs (red) incubated with DiO-labeled TCMK-1 cells (green) for 0 h or 4 h. **e** Western blot analysis of the protein levels of KIM-1 and LCN-2 in TCMK-1 cells incubated with or without C-EVs or COM-EVs treated with or without COM for 24 h. TCMK-1 cells without any treatment were utilized as a negative control, whereas those exposed to COM for 24 h were designated as the positive control. Ctrl: control group, COM: TCMK-1 cells with COM stimulation for 24 h, C-EVs: TCMK-1 cells with C-EVs stimulation for 24 h, COM-EVs: TCMK-1 cells with COM-EVs stimulation for 24 h, COM + C-EVs: TCMK-1 cells with COM and C-EVs stimulation for 24 h, COM + COM-EVs: TCMK-1 cells with COM and COM-EVs stimulation for 24 h. **f** Relative quantification of the data presented in (**e**). **g** Volcano plot of differentially expressed protein in COM-EVs and C-EVs (protein upregulated in red and protein downregulated in blue). **h** Schematic diagram of the experimental procedure for investigating the mechanisms underlying COM-EVs on the phenotypes of TCMK-1 cells. **i** Western blot analysis of the protein levels of KIM-1 and LCN-2 in TCMK-1 cells incubated with COM-EVs from BMDM transfected with scrambled siNC or siGRP94. All the data are presented as means ± SEM. Significance was determined by one-way ANOVA with Tukey’s multiple comparisons test, *n* = 3 biologically independent samples, **p < 0.01, *p < 0.05

To investigate whether EVs from BMDMs with COM stimulation affect surrounding renal tubular cells and subsequently affect kidney stone formation, we frist characterized EVs in vitro*.* TEM and NTA analysis revealed that both C-EVs and COM-EVs exhibited a typical bilayer lipid membrane structure, with an average diameter of approximately 170 nm (Fig. 2a-b). These EVs positively expressed EVs marker (Alix, TSG101 and CD63), and were negative for Golgi marker (GM130) (Fig. 2c). EVs exert regulatory effects on target cells by transferring bioactive substances; hence, the ingestion of EVs by recipient cells is an essential process [32]. Therefore, we first evaluated whether EVs could be taken up by TCMK-1 (a mouse tubular epithelial cell line) in vitro. Confocal images revealed positive signals (red) of EVs in TCMK-1 cells (green), indicating that EVs were phagocytized by TCMK-1 cells (Fig. 2d). Next, we examined whether EVs from BMDMs stimulated with COM were involved in the development of KSD. In vitro western blot analysis demonstrated an increase in the expression of classic injury markers, including LCN-2 (lipocalin-2) and KIM-1 (kidney injury molecule 1), indicating that EVs from COM-stimulated BMDMs promoted TCMK-1 injury (Fig. 2e-f). To clarify the functional relevance of macrophage-derived EVs, we applied siRNA to specifically silence of Rab27a in BMDMs, as Rab27a is known to participate in EVs release [33]. We then employed the transwell system to explore the role of BMDMs-derived EVs on TCMK-1 cells. Rab27a knockdown macrophages stimulated with CaOx crystals were placed in the upper chamber, while TCMK-1 cells were in the lower chamber. Our findings showed that Rab27a knockdown in macrophages reduced the release of EVs (Fig. S2a-b) and mitigated injury to TCMK-1 cells compared to the control group. This is evidenced by significantly lower levels of the key injury markers KIM-1 and LCN-2 (Fig. S2c). These indicated that inhibiting EVs secretion from macrophages under CaOx crystal stimulation can alleviate tubular injury.

Increasing evidence indicates that EVs can impact the functions of recipient cells by delivering multiple types of bioactive cargos, especially microRNAs (miRNAs) and proteins. To explore the underlying mechanism by which COM-EVs promote kidney stones, we analyzed the differences in protein expression between C-EVs and COM-EVs using proteomics. We identified 194 differentially expressed proteins between C-EVs and COM-EVs group, with most of the upregulated differential proteins enriched in protein processing in ER (Fig. S3a-b). Notably, we observed an elevation in glucose-regulated protein 94 (GRP94) levels (Fig. 2g). Further in vitro validation revealed that CaOx-stimulating macrophages excreted more GRP94-containing EVs (Fig. S3c). To validate GRP94 as a critical component of macrophage-derived EVs mediating tubular injury, we knocked down the GRP94 in BMDMs using siRNA (Fig. S3d), then collected the EVs, and used them to intervene with TCMK-1 cells (Fig. 2h). Western blot analysis showed that, compared with the control group, the levels of KIM-1 and LCN-2 in TCMK-1 cells were significantly reduced in the GRP94 knockdown group (Fig. 2i). These results suggest that EVs containing GRP94, produced by COM-stimulated BMDMs, may be taken up by surrounding renal tubular epithelial cells, thereby promoting kidney injury.

### EVs from BMDMs with COM promote kidney stone formation in mouse

**Fig. 3:**
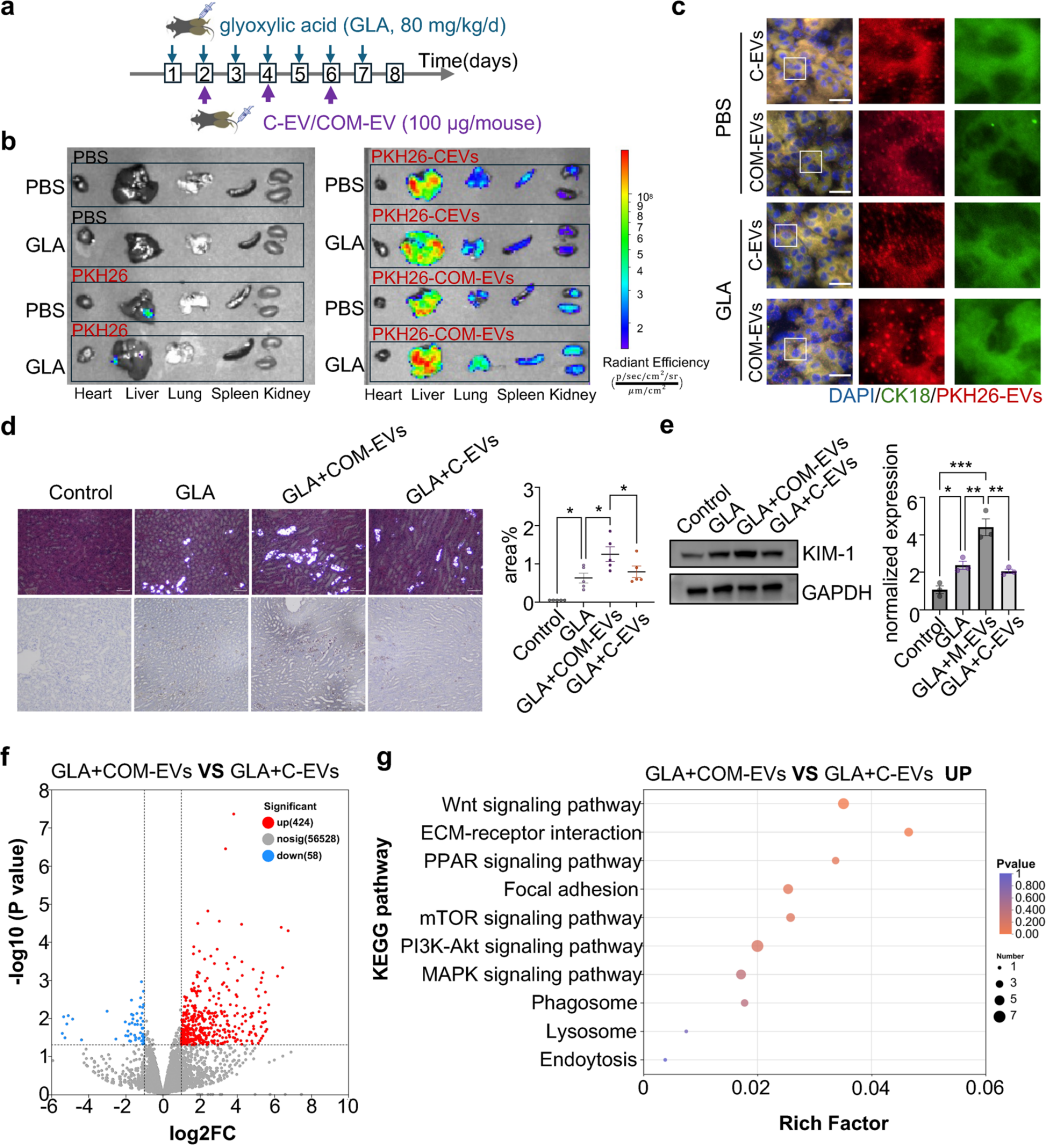
EVs from BMDMs with COM stimulation promote CaOx crystals deposition in the kidney. **a** Schematic diagram of the experimental procedure for investigating the role of EVs in the development of KSD. **b** Representative in vivo imaging system (IVIS) images of organs harvested from mice at 4 h after intravenous tail injection of PKH26-labeled EVs. Mice that received PKH26 dye alone were included as negative controls. **c** Fluorescence images of PKH26-EVs in CK18–labeled kidney. **d** Representative H&E staining observed under a polarizing microscope and Von Kossa staining viewed with a light microscope of the kidneys (left), along with statistical analysis of the CaOx crystal quantities from the H&E staining (right). **e** Western blot analysis of the protein levels of KIM-1 in BMDMs treated with or without COM for 24 h. Relative quantification (left) of the data presented in (right). **f** Volcano plot of differentially genes in GLA + COM-EVs and GLA + C-EVs (genes upregulated in red and genes downregulated in blue). **g** KEGG enrichment map of RNA sequencing in mouse kidneys. All the data are presented as means ± SEM. Significance was determined by one-way ANOVA with Tukey’s multiple comparisons test, ***p < 0.001, **p < 0.01, *p < 0.05

To investigate the impact of EVs from BMDMs with COM on kidney stone formation in vivo, we injected PKH26 (a lipophilic fluorescent dye)-labeled EVs into a GLA reduced mouse model via tail vein injection (Fig. 3a). Consistent with a previous report [34], our study revealed that EVs accumulated mainly in the heart, liver, lungs, spleen, and kidney (Fig. 3b). In the kidney, EVs were engulfed by renal tubular epithelial cells (CK18-positive). Immunofluorescence showed that EVs could be taken up by renal epithelial cells (Fig. 3c). Analysis of mouse kidney sections revealed that, compared with C-EVs group, COM-EVs exacerbated the deposition of CaOx crystals (Fig. 3d) and increased the expression of KIM-1 (Fig. 3e). In addition, kidneys treated with EVs underwent RNA sequencing (RNA-seq). In the GLA-administered group, we identified 424 differentially expressed genes between the C-EVs group and COM-EVs group (Fig. 3f). COM-EVs showed upregulation of many pathways closely associated with tubular injury, including the PPAR signaling pathway and MAPK signaling pathway, as well as pathways related to cell adhesion (Fig. 3g). These results suggested that EVs from COM-stimulated BMDMs promoted tubular epithelium injury and incresed the deposition of CaOx crystals in the mouse kidney.

### COM-induced ERS mediates BMDMs lysosomal dysfunction and EVs release

**Fig. 4:**
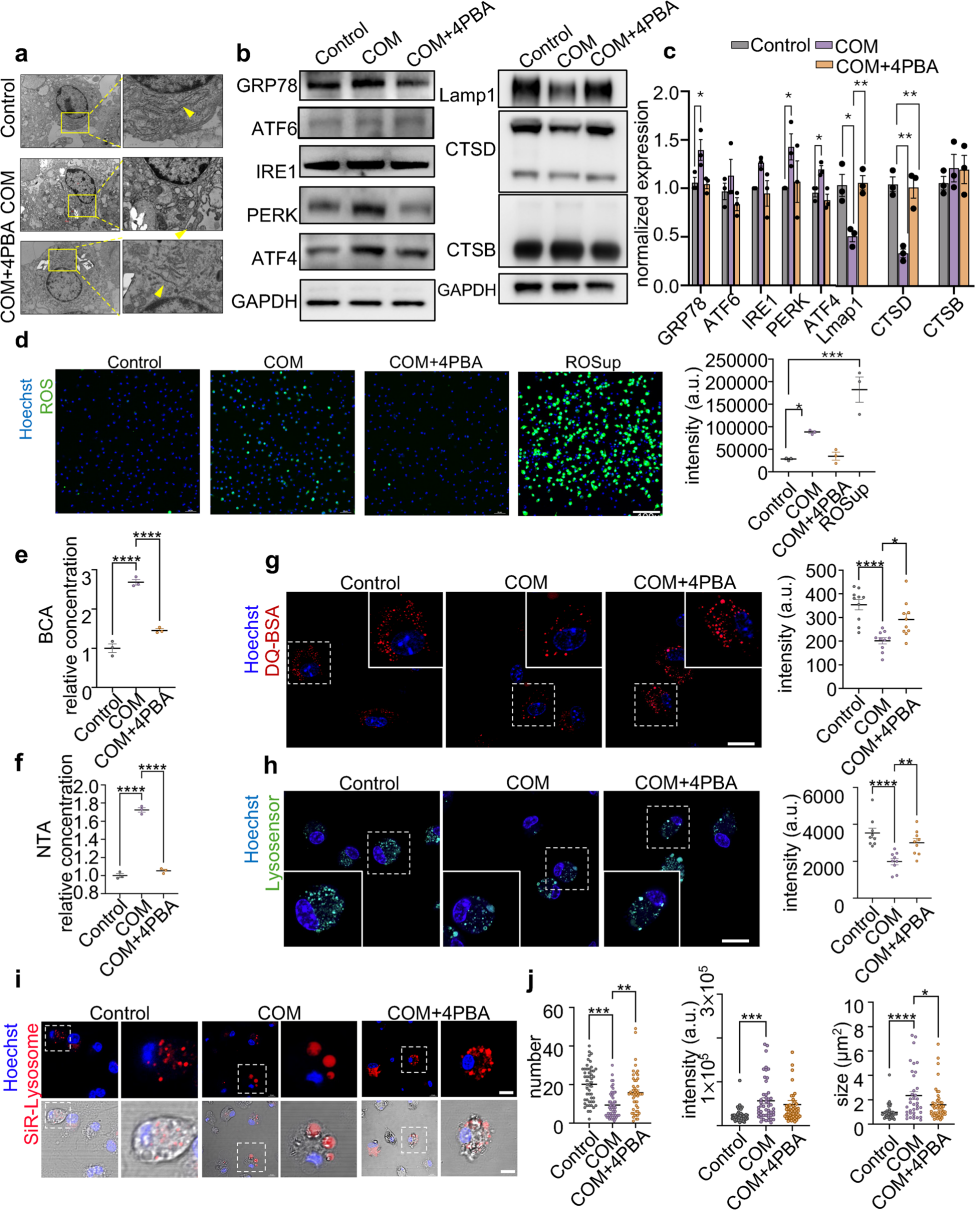
COM-induced ERS mediates BMDM lysosomal dysfunction and EVs release. **a** TEM images of the ER in BMDMs treated with COM in the absence or presence of 4PBA for 24 h. Untreated BMDMs served as controls. The yellow arrow indicates the ER. **b**-**c** Western blot analysis of the ERS-related (GRP78, ATF6, IRE1, PERK, and ATF4) and lysosome-related (Lamp1, CTSD, CTSB) protein levels in BMDMs treated with COM in the absence or presence of 4PBA for 24 h. Untreated BMDMs served as controls. Relative quantification (**c**) of the data presented in (**b**). **d** Representative fluorescence images and quantitative analysis of ROS in BMDMs. Relative quantification (left) of the data presented in (right). **e–f** BCA or NTA analysis of the concentration of EVs. **g**-**j** To detect lysosome function, BMDMs were treated with COM in the absence or presence of 4PBA for 24 h. Untreated BMDMs served as controls. **g** Representative fluorescence images of DA-BSA (Red) staining detected via laser scanning confocal microscopy. Relative quantification (left) of the data presented in (right). **h** Representative fluorescence images of Lysosensor (green) staining detected via laser scanning confocal microscopy. Relative quantification (left) of the data presented in (right). **i-j** Representative fluorescence images of SiR-Lysosome (red) staining detected via laser scanning confocal microscopy (**i**) and the quantification of the number, size, and fluorescence intensity of red (**j**). All the data are presented as means ± SEM. Significance was determined by one-way ANOVA with Tukey’s multiple comparisons test, *n* = 3 biologically independent samples, ****p < 0.0001, ***p < 0.001, **p < 0.01, *p < 0.05

Given that we have established the role of macrophage-released EVs in kisney stone formation, we next determined how CaOx crystal invasion induces EVs release. The proteomic sequencing results of EVs suggest that their origin may be related to ER. Additionally, in the extracellular matrix of blood vessel walls, the deposition of calcium phosphate crystals ERS induces vascular calcification by promoting EVs release [35]. Therefore, we hypothesize that crystal-stimulated EVs release is mediated by ERS. To validate this hypothesis, we first examined the ER status in macrophages following crystal stimulation. TEM images showed that the laminar distribution of the ER expanded due to the presence of COM (Fig. 4a). Further evidence from western blot analysis indicated an upregulation of ERS-related proteins, including PERK, ATF4, and GRP78 (Fig. 4b-c). Simultaneously, COM stimulation induced the accumulation of intracellular reactive oxygen species (ROS) (Fig. 4d and S4). These results suggest that COM stimulation causes increased ERS in BMDMs. Additionally, we used 4-phenylbutyric acid (4PBA), an ERS inhibitor [36], to suppress ERS following COM stimulation. As expected, treatment with 4PBA inhibited ERS, a as demonstrated by the restored dilation of the ER, the effective reduction in the increases of PERK, ATF4, and GRP78 expression (Fig. 4b-c), and decreases the intensity of ROS (Fig. 4d and S4). Notably, inhibition of ERS reduced EVs release from macropahges with COM stimulation (Fig. 4e-f). Taken together, these results demonstrate that EVs release following COM stimulation is mediated by ERS.

We then investigated how ERS induced by crystal invasion affects the release of EVs. Multivesicular bodies can fuse with lysosomes to form phagolysosomes, which degrade EVs and regulate their production [37]. However, it remains unclear whether ERS caused by crystal invasion controls EVs release by regulating lysosomal function. To address this, we examined whether ERS was triggered and could regulate lysosomal function in infected macrophages. First, we assessed changes in lysosomal-related proteins using western blotting and found that crystal stimulation led to a decrease in the expression of these proteins, including Lamp1, CTSD and CTSB. Treatment with 4PBA improved these changes (Fig. 4b-c). Additionally, we assessed total lysosomal proteolytic activity using DQ-BSA (dye-quenched bovine serum albumin), a fluorescent probe that enables quantitative assessment of lysosomal degradation efficiency [38, 39]. Fluorescence imaging results indicated that lysosomal function was rescued by 4PBA (Fig. 4g). Further evidence was provided by Lysosensor, a pH-sensitive fluorescent probe (Fig. 4h). The enzymatic activity of CTSD was measured using SiR-lysosome, which is based on pepstatin A (a CTSD-binding peptide) labeled with the proprietary fluorophore silicon rhodamine (SiR) [39]. Treatment with 4PBA alleviated the increase in lysosomal volume of BMDMs caused by COM crystals, as demonstrated by fluorescence images and intensity values (Fig. 4i-j).

Thus, these data suggested that ERS induced by crystal invasion negatively impacts lysosomal function, thereby influencing the release of EVs.

### PERK-mediated BMDMs lysosomal dysfunction induces EVs release

**Fig. 5:**
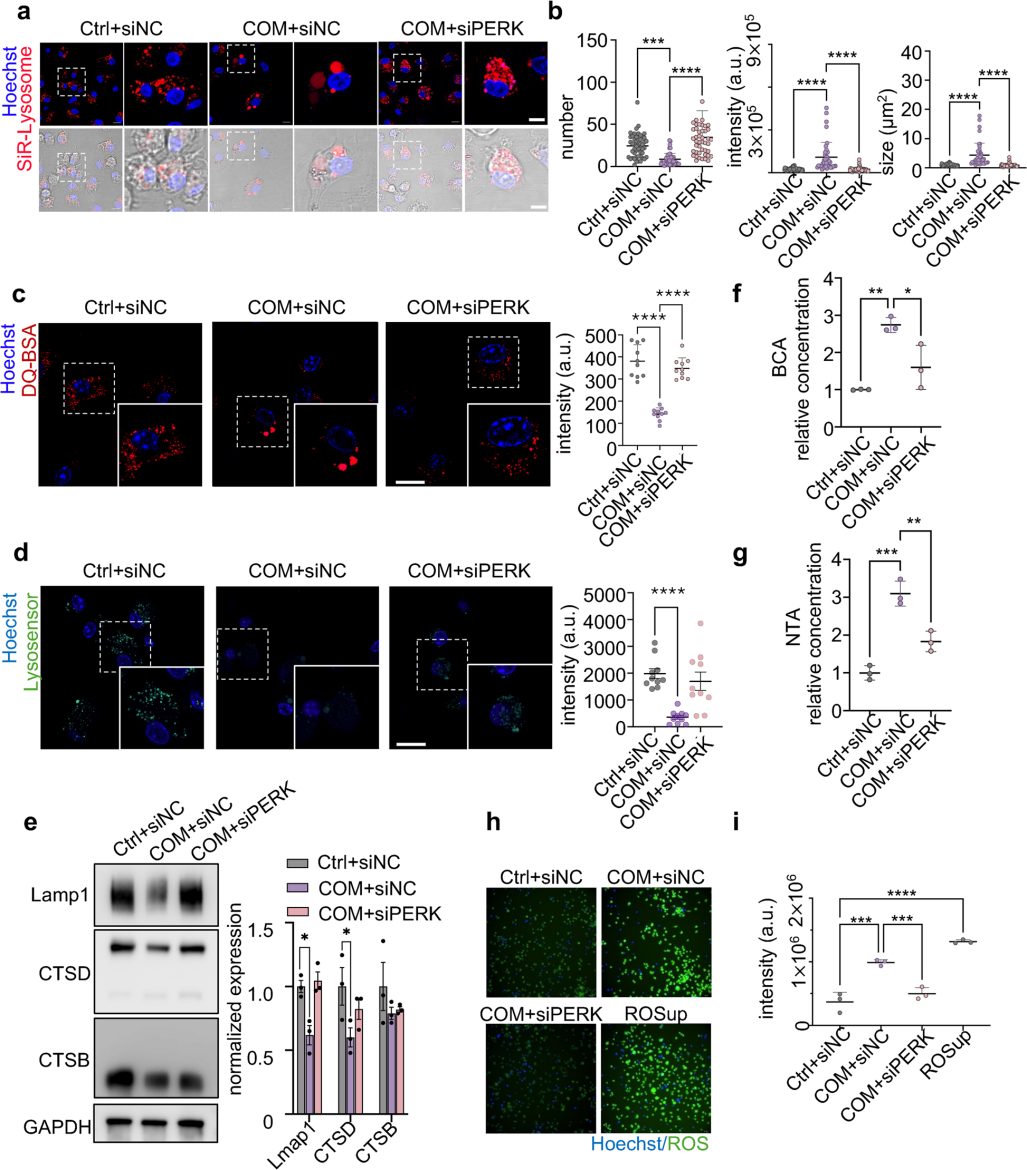
PERK-mediated lysosomal dysfunction induces EVs release during COM stimulation. **a-d** To detect lysosome function and EVs release, BMDMs were transfected with scrambled siRNA (siNC) or PERK siRNA (siPERK) and then treated with COM for 24 h. BMDMs transfected with siNC alone served as controls. **a-b** Representative fluorescence images of SiR-Lysosome (red) staining detected via laser scanning confocal microscopy (**a**) and the quantification of the number, size, and fluorescence intensity of red (**b**). **c** Representative fluorescence images of DA-BSA (Red) staining detected via laser scanning confocal microscopy. Relative quantification (left) of the data presented in (right). **d** Representative fluorescence images of Lysosensor (green) staining detected via laser scanning confocal microscopy. Relative quantification (left) of the data presented in (right). **e** Western blot analysis of the ERS-related protein levels of Lamp1, Cathepsin D, Cathepsin B. Relative quantification (left) of the data presented in (right). **f-g** BCA and NTA analysis of the concentration of EVs**. h-i** Representative fluorescence images and quantitative analysis of ROS in BMDMs. Relative quantification (**i**) of the data presented in (**h**). All the data are presented as means ± SEM. Significance was determined by one-way ANOVA with Tukey’s multiple comparisons test, *n* = 3 biologically independent samples, ****p < 0.0001, ***p < 0.001, **p < 0.01, *p < 0.05

After determining that ERS induced by COM stimulation caused lysosome dysfunction, we further focused on the the PERK signaling pathway to identify the specific mechanism affecting EVs release. On our results showed upregulated PERK expression after infection (Fig. 4b). To verify the role of PERK in this mechanism, we employed small interfering RNA targeting PERK (siPERK) and the PERK inhibitor GSK2606414 (GSK) to inhibit PERK expression. Following treatment with siPERK, the expression of PERK and activation of the downstream ATF4 pathway were significantly reduced in COM-stimulated BMDMs (Fig. S5). As expected, the enlargement of lysosomal cluster was also alleviated (Fig. 5a-b and Fig. S6a-b), and lysosomal function was restored to the normal levels (Fig. 5c-d and Fig. S6c-d) with the reduction in PERK expression. Moreover, the inhibition of PERK by siRNA or GSK was accompanied by a decrease in lysosome-related protein, including lamp1, CTSD and CTSB (Fig. 5e and Fig. S6e-f). Thes results showed that lysosomal function was partially recovered, indicating that lysosomal damage is PERK-dependent and due to direct CaOx toxicity. Notably, the secretion of EVs was significantly decreased following PERK inhibition (Fig. 5f-g and S6g-h).

Additionally, high levels of ROS were reduced following the inhibition of PERK using either siRNA or GSK (Fig. 5h-i and S6i). To identify the molecular mechanism of PERK actication following CaOx exposure, we used Tempol, a potent ROS-neutralizing agent, to inhibit ROS levels [40]. The results indicated that BMDMs treated with both Tempol and COM exhibited a significant decrease in the expression of PERK and ATF4 proteins compared to those treated with COM alone (Fig. S7a-b). These findings suggest a bidirectional relationship between CaOx-induced PERK activation and ROS generation. Overall, these results confirmed that PERK induced lysosomal dysfunction in COM-stimulated BMDMs, triggering EVs release. This suggests that the PERK pathway plays a critical role in regulating EV release in response to crystal-induced stress.

**Fig. 6:**
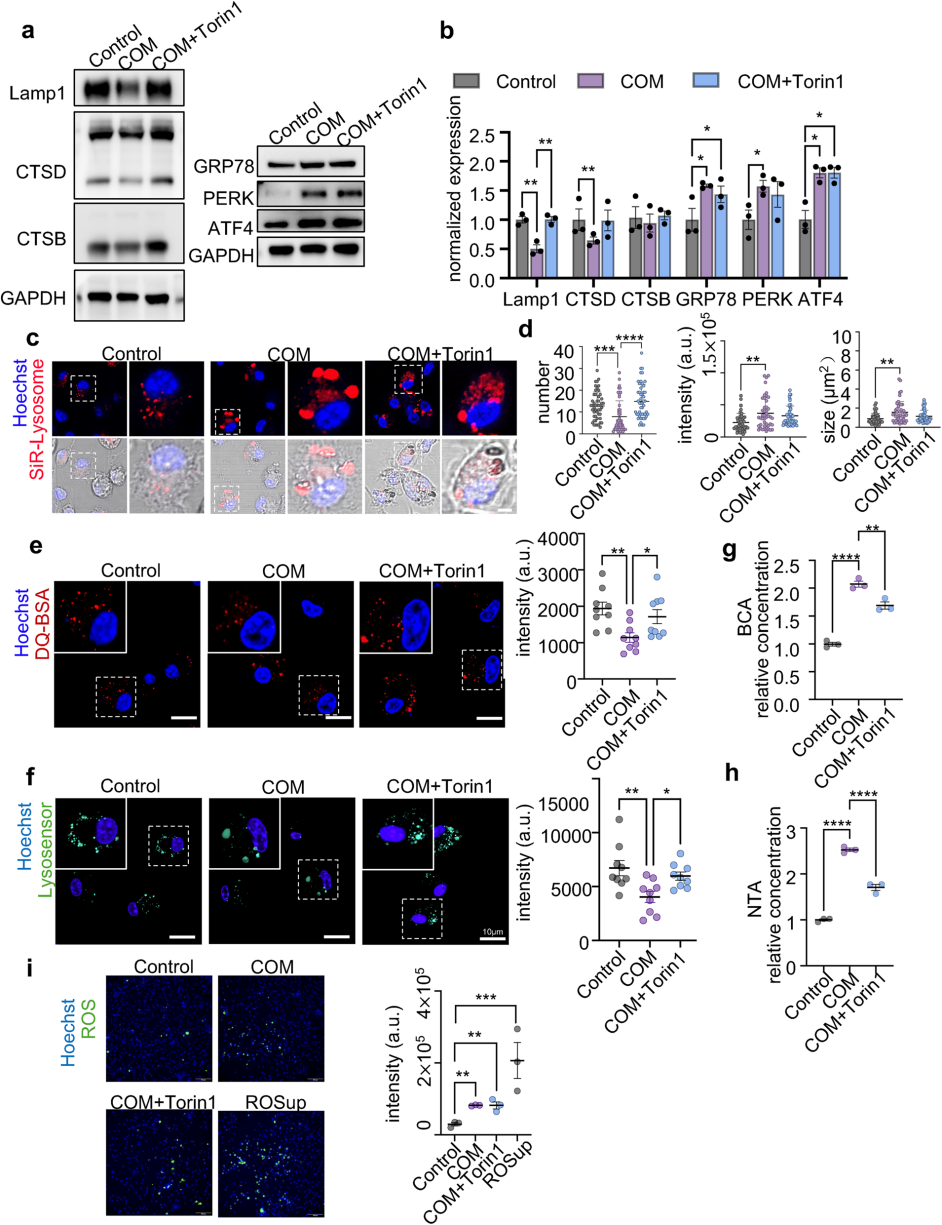
Lysosomal dysfunction of BMDMs induced by COM stimulation. **a-b** Western blot analysis of the lysosome-related (Lamp1, Cathepsin D, Cathepsin B) and PERK pathway-related (GRP78, PERK, and ATF4) protein levels in BMDMs treated with COM in the absence or presence of Torin1 for 24 h. Untreated BMDMs served as controls. Relative quantification (**b**) of the data presented in (**a**).** c-d** Representative fluorescence images of SiR-Lysosome (red) staining detected via laser scanning confocal microscopy (**c**) and the quantification of the number, size, and fluorescence intensity of red (**d**). **e** Representative fluorescence images of DA-BSA (Red) staining detected via laser scanning confocal microscopy. Relative quantification (left) of the data presented in (right). **f** Representative fluorescence images of Lysosensor (green) staining detected via laser scanning confocal microscopy. Relative quantification (left) of the data presented in (right). **g** Representative fluorescence images and quantitative analysis of ROS in BMDMs. Relative quantification (left) of the data presented in (right). **h-i** BCA and NTA analysis of the concentration of EVs. All the data are presented as means ± SEM. Significance was determined by one-way ANOVA with Tukey’s multiple comparisons test, *n* = 3 biologically independent samples, ****p < 0.0001, ***p < 0.001, ** *p* < 0.01; ns = not significant

To further demonstrate this cascade, we restored lysosomal function using Torin1, a lysosomal biogenesis inducer [41], to observe changes in PERK signaling pathway and the release of EVs. Western blot analysis revealed that while Torin1 treatment enhanced the expression of lysosome-related proteins, including Lamp1, CTSD and CTSB (Fig. 6a-b). Additionally, immunofluorescence analyses showed that lysosomal function was increased in COM-stimulated BMDMs treated with Torin1 compared to those that were only COM-stimulated (Fig. 6c-f). These results showed that Torin1 recused the function of lysosome. Simultaneously, we used a plasmid to overexpress TFEB to specifically assess the role of lysosomal rescue. Western blot analysis confirmed that the level of Lamp1 was increased following TFEB overexpression (Fig. S8a). DQ-BSA images indicated reduced lysosomal acidity in BMDMs after TFEB overexpression (Fig. S8b). Furthermore, we observed both PERK knockdown and TFEB overexpression resulted in similar degrees of rescue of lysosomal enzyme activity (Fig. S8c). Importantly, BCA and NTA analyses revealed that the restoration of lysosomal function decreased EVs release from COM-stimulated BMDMs thourgh Torin-1 treatment or TFEB overexpression (Fig. 6g-h, and Fig. S8d). Therefore, these results suggest that the release of EVs induced by COM is regulated by lysosomal function. We further explored the impact of lysosomal rescue on the PERK pathway. However, treatment with Torin1 did not reduce the expression of PERK signaling pathway-related proteins (GRP78, PERK and ATF4) (Fig. 6a-b). Similarly, the release of ROS could not be inhibited (Fig. 6i). These findings indicate that restoring lysosomal function does not alter the activation of the PERK pathway induced by COM.

**Fig. 7:**
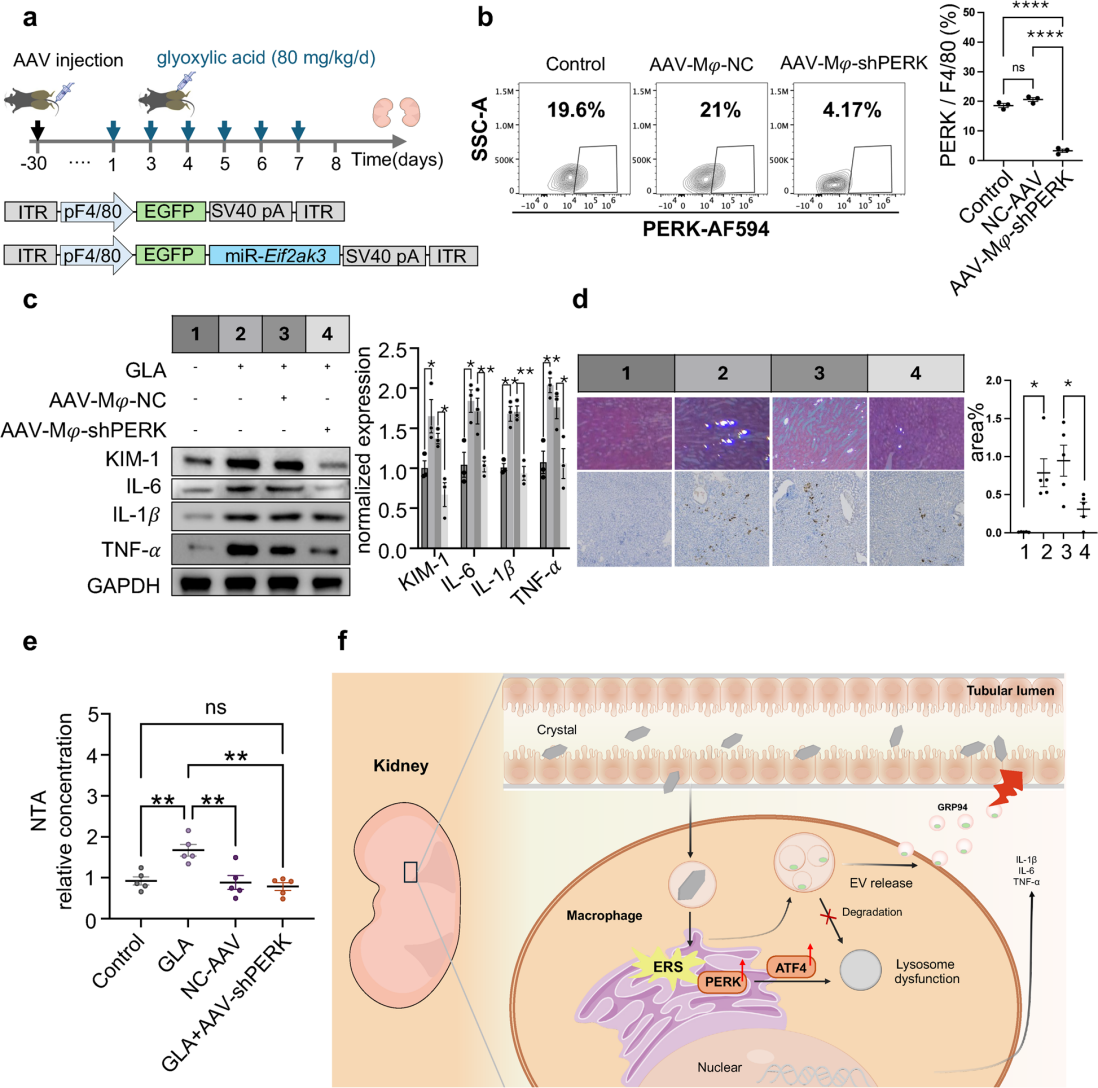
Knockdown of PERK in macrophages in vivo alleviates renal calcium crystals deposition. **a** Schematic diagram of the experimental procedure used to investigate the role of PERK in macrophages in the development of CaOx crystal deposition. The mice were randomly divided into 4 groups. The first two groups were pretreated with AAV-shPERK or with a scrambled control AAV (NC-AAV) for 30 d. The remaining two groups received an equivalent volume of saline solution for 30 d as a control. Next, the mice in the AAV-shPERK and control groups were intraperitoneally injected with 80 mg/kg/d glyoxylic acid (GLA) for 7 days. The mice were culled on Day 8 for further analysis. *n* = 5 mice. **b** FCM staining analysis of kidney macrophages from mice treated with NC-AAV or AAV-shPERK. Relative quantification (right) of the data presented in (left). **c** Western blot analysis of the protein levels of KIM-1, TNF-α, IL-6 and IL-1β in kidney. Relative quantification (right) of the data presented in (left). **d** Representative H&E staining observed under a polarizing microscope and Von Kossa staining viewed with a light microscope of the kidneys (left), along with statistical analysis of the CaOx crystal quantities from the H&E staining (right). Gourp1: control group; Group2: mice were intraperitoneally injected with 80 mg/kg/d glyoxylic acid (GLA) for 7 days; Gourp3: mice were pretreated with a scrambled control AAV (NC-AAV) for 30 d, then were intraperitoneally injected with 80 mg/kg/d glyoxylic acid (GLA) for 7 days; Group4: mice were pretreated with AAV-shPERK for 30 d, then were intraperitoneally injected with 80 mg/kg/d glyoxylic acid (GLA) for 7 days. **e** NTA analysis of the concentration of EVs in urine of mice. All the data are presented as means ± SEM. Significance was determined by one-way ANOVA with Tukey’s multiple comparisons test, ****p < 0.0001, ***p < 0.001, *p < 0.05. **f** Schematic representation of a pathogenic mechanism by which macrophage regulates CaOx crystal deposition in the kidney. After CaOx crystal stimulation, ERS is triggered, activating the PERK signaling pathway. This leads to lysosomal dysfunction and the release of a large amount of EVs that transmit signals to promote renal tubular injury, ultimately leading to CaOx crystal formation in the kidney

Finally, we determined the role of PERK pathway in lysosomal dysfunction-induced EVs release and kidney stone formation in vivo. To decrese the activation of PERK on macrophages, we used a macrophages cell-specific adeno-associated virus (AAV) targeting approach to knock down PERK (*Eif2ak3*) in mouse by injecting mice via the tail vein with an adeno-associated virus carrying PERK shRNA (AAV-shPERK), which targets macrophages using the F4/80 promoter (Fig. 7a). FCM and immunofluorescence staining analyses showed that AAV-shPERK treatment did not significantly alter the number of renal macrophages (F4/80^+^) compared to control groups (Fig. S9a). Furthermore, there were no notable changes in the expression of macrophage activation markers, including iNOS, and TLR4, following AAV-shPERK administration (Fig. S9b). Importantly, this strategy successfully reduced the expression of PERK in macrophages within the kidney, as confirmed by FCM analysis of F4/80^+^ cells isolated from kidney tissues (Fig. 7b). These results collectively demonstrate that AAV-shPERK selectively targets PERK in macrophages without broadly affecting their abundance or activation state in the renal microenvironment. Knockdown of PERK in macrophages (GLA + AAV-shPERK) inhibited stone formation in the kidneys compared to the model group (GLA) and significantly reduced the level of inflammatory and damage-related proteins in the kidneys (Fig. 7c-d). Futuremore, the results revealed that kidneys with reduced PERK expression in macrophages produced fewer proinflammatory cytokines in response to hyperoxaluria than kidneys with intact macrophages (Fig. S10). Importantly, NTA results showed that knockdown of PERK in macrophages reduced the concentration of EVs in the urine of mice with the stone model (Fig. 7e). These observations emphasize the role of PERK in macrophages, which may contribute to injury due to crystal deposition and secondary inflammation (Fig. 7f).

## Discussion

In this study, we found that CaOx crystal-stimulated BMDM-EVs secretion is a key factor in KSD and explored the underlying mechanisms governing this secretion. Our findings elucidate a crucial step in crystal-induced inflammatory changes, shedding light on the mechanisms of renal injury during crystal exposure and establishing a framework for understanding how phagocytes contribute to kidney damage. Additionally, we suggest that inhibiting ERS through PERK knockdown in macrophages may offer a viable strategy for treating renal CaOx crystal deposition. Furthermore, crystals measuring 3–5 μm in size represent an early stage of kidney stone formation, during which macrophages amplify and spread damage to neighboring cells, ultimately leading to stone formation. This critical stage presents a significant opportunity for intervention in the development of kidney stones.

Notably, our findings add nuance to the dual role of macrophages in KSD. They release harmful EVs that exacerbate injury to renal tubules and promote calcification within them. This suggests that the functional plasticity of macrophages influences their impact on stone pathogenesis. Similarly, recent studies have also shown that exosomes from CaOx-treated macrophages can induce apoptosis in renal cells and activate fibroblasts [42–44]. These findings highlight the ongoing debate regarding whether BMDM-EVs act as "participants" or "rescuers" in the development of KSD. This question warrants further exploration in future studies.

Our work also highlights GRP94 as a critical mediator within COM-derived EVs that promote tubular injury. Proteomic analysis revealed significant enrichment of GRP94 in EVs from COM-stimulated macrophages, and knockdown of GRP94 ameliorated EV-induced injury in renal tubular cells. This finding aligns with emerging evidence that ERS-related proteins packaged into EVs can propagate inflammatory and injury signals across tissues. While GRP94 appears to be a key effector, it is likely that other EV cargo components (e.g., miRNAs, lipids) also contribute to the observed phenotypes. Future studies using high-sensitivity profiling should aim to comprehensively characterize the bioactive molecules within EVs and clarify their individual roles in crystal nucleation and adhesion. The significant attenuation of pro-apoptotic effects following GRP94 knockdown suggests that ERS-related cargo in EVs may act as key mediators of kidney injury in nephrolithiasis.

The selective incorporation of ER proteins into EVs may involve several interconnected mechanisms [45]. Membrane contacts sites (MCSs) between the ER and organelles like endosomes play a crucial role in facilitating cargo selection and loading by enabling interactions with sorting machinery, such as the ESCRT (endosomal sorting complexes required for transport) system [46, 47]. Additionally, ER-associated proteins can influence the dynamics of these MCSs, thereby regulating the sorting of specific proteins and RNAs into intraluminal vesicles (ILVs). Autophagy-related proteins, particularly LC3, also contribute to this process by promoting the loading of cargo into EVs during stress conditions, suggesting that cellular stress can enhance the recruitment of ER proteins into EVs [48–50]. Furthermore, the remodeling of ER membranes under various physiological states may provide additional pathways for the integration of these proteins into EVs, highlighting the complex interplay between ER dynamics, cargo selection, and EV biogenesis [51].

A key finding of our study is the central role of PERK-mediated ERS in promoting lysosomal dysfunction and subsequent EVs secretion in macrophages following CaOx crystal exposure. While ERS has been previously implicated in vascular calcification and EVs release in other contexts, its role in macrophage responses during nephrolithiasis had not been elucidated. Our data show that COM crystals specifically activate the PERK-TF4 branch of the unfolded protein response, distinct from IRE1 or ATF6 pathways, leading to defective lysosomal acidification and proteolytic activity. This lysosomal impairment inhibits the degradation of multivesicular bodies, thereby facilitating EVs secretion-a phenomenon supported by studies in other disease models. Importantly, inhibition of PERK, either genetically or pharmacologically, restored lysosomal function, suppressed EVs release, and attenuated crystal deposition in vivo, underscoring the therapeutic potential of targeting PERK in macrophages.

In our study, the results indicate that CaOx stimulation induces ERS, activates PERK, and causes lysosomal damage. Inhibition of PERK alleviates ERS while restoring lysosomal function. However, we observed that Torin1 can rescue lysosomes but does not improve ERS. These findings suggest that PERK activation influences lysosomal damage through one of its pathways. Other studies also report that crystals can directly damage lysosomes; for instance, a study by Chrisovalantis Papadopoulos et al. highlights this effect [52]. The damage to lysosomes caused by crystals arises from two main aspects: first, the crystals directly induce lysosomal damage; second, they trigger oxidative stress, which, through the activation of the ERS response, further promotes lysosomal damage. When CaOx are relatively small, lysosomes can degrade them, and oxidative stress is the primary pathway leading to lysosomal damage. However, when the crystals are larger, lysosomes cannot degrade them, resulting in both oxidative stress and the crystals themselves contributing to lysosomal damage.

Despite these promising findings, several limitations must be acknowledged. First, we focused primarily on Mφ-EVs, while EVs derived from other cell types (e.g., tubular epithelial cells) may also contribute to stone formation [53]. A more comprehensive EV profiling across kidney cell types could provide a holistic view of intercellular communication in KSD. Second, all animal experiments were conducted in male mice to exclude the influence of estrogen, which is known to protect against stone formation [54]. Consequently, our findings may not fully represent female pathophysiology. Future studies should include both sexes to evaluate potential sex-specific mechanisms. Third, although we observed systemic accumulation of injected EVs in the liver, the functional implications of hepatic EV uptake in the context of KSD remain unexplored. Given the liver's role in systemic inflammation and metabolism, future work should investigate possible liver–kidney crosstalk mediated by EVs.

## Conclusion

The results of this study revealed that macrophages underwent ERS after crystal stimulation via the PERK signaling pathway, leading to lysosomal swelling, decreased function, and ultimately promoting EVs release. These EVs exacerbate renal tubular epithelial cell damage and inflammation to promote CaOx crystal deposition. Thus, we identified a previously unknown mechanism by which macrophages are affected by crystal stimulation (Fig. 7f).

## Materials and methods

### COM crystal preparation and characterization

Briefly, 20 mL of 2-mM CaCl_2_ solution was quickly mixed with 20 mL of 1 mM CaOx crystal mixture, incubated (10 rpm) for 2 h, and then centrifuged. The solid product underwent a double rinse with anhydrous methanol, followed by drying, yielding COM particles sized between 3–6 μm [55]. The samples were placed on a specimen holder and coated with gold powder. Next, the samples were imaged using an SEM system (ZEISS EVO 10, Germany) at magnifications of 1000 × and 3000 ×.

Completely dried samples of ~ 2.5 mg of COM crystals were mixed with 250 mg of KBr, ground to a powder, pressed, and scanned over the 4000–400 cm^−1^ wavenumber range.

### BMDMs isolation and culture

BMDMs were isolated from the tibias and femurs of 8-week-old male C57BL/6J mice and cultured in DMEM (Gibco, USA) mixed with 10% fetal bovine serum (Gibco), 50 U/mL penicillin, 50 μg/mL streptomycin, and 20 ng/mL murine macrophage colony-stimulating factor (Novoprotein, Shanghai, China) for 6 d [56]. Mature BMDMs were used for further experiments (Fig. S1c-e). BMDMs were stimulated with COM at a ratio of 3–5 µm COM particles per macrophage.

When indicated, BMDMs were treated with 1 μM Torin1 (MedChemExpress, NJ, USA), 5 mM 4PBA (MedChemExpress), 0.3 mM Tempol (MedChemExpress) or 500 nM GSK2606414, which is a PERK inhibitor (Selleckchem, Shanghai, China).

### siRNA and transfection

BMDMs were subjected to transfection with 20 nM siRNA targets PERK or 40 nM siRNA targets GRP94 (HIPPOBIO, Huzhou, China) that using an siRNA transfection reagent (NanoTrans20; HIPPOBIO, Huzhou, China). Following transfection, the cells were treated 48 h post-transfection. The resulting transfected cells were subsequently utilized in further experiments.

### Plasmids and transfection

BMDMs were transfected with 2.5 µg of TFEB plasmids using Lipofectamine 3000 (Invitrogen) according to the manufacturer's instructions. After transfection, the cells were incubated for 48 h before being utilized in subsequent experiments.

### Transmission electron microscopy (TEM)

An EVs-containing suspension was applied to a 200-mesh carbon-coated copper grid for 5 min to allow absorption, after which the surplus was wiped away. 1% phosphotungstic acid staining was performed for 1–2 min at room temperature. A JEM-1400FLASH transmission electron microscope was used for observation.

Post-fixation of the cells with 1% osmium tetroxide followed the initial fixation with 3% glutaraldehyde. Subsequent dehydration was performed using a graded acetone series, prior to infiltration with Epox 812. For the semithin sections, staining was achieved with methylene blue, while the ultrathin sections, obtained by cutting with a diamond knife, were treated with a combination of uranyl acetate and lead citrate for staining.

### DQ-BSA and Lysosensor fluorescence measurement

To measure lysosomal acidity, BMDMs were cultured in serum-free medium with 0.1 mg/mL DQ-BSA (Thermo Fisher, Waltham, MA) or 1 μM LysoSensor DND-189 (Yeasen, shanghai, China). After the cells were washed with PBS, the fluorescence intensity of DQ-BSA or LysoSensor was measured with a confocal laser scanning microscope (CLSM, Nikon, TiA1-N-STORM, Japan).

### SiR-lysosome fluorescence analysis

BMDMs with or without COM stimulation were incubated with 1 μM SiR-lysosome reagent (CY-SC012, Cytoskeleton, Denver, USA) for 1 h at 37°C. After the samples were washed with PBS, Hoechst 33342 was added, and the mixture was incubated for 5 min at room temperature. The fluorescence intensity of the SiR-lysosome system was measured using CLSM (TiA1-N-STORM, Nikon, Japan). SiR-lysosome intensity was quantified with the same settings for the SiR-lysosome channel, including laser power, exposure, and gain, and the pixel intensity remained below saturation. The average intensity of the images of the channels corresponding to the SiR-lysosome signal in the selected region was measured using confocal laser scanning microscopy.

### Western blotting

The samples of cell or EVs were lysed in RIPA buffer (Beyotime, Shanghai, China) containing phosphatase and protease inhibitors for 30 min. The total protein concentration was determined using a BCA kit (P0010, Beyotime). Equal amounts of protein were subjected to 8–12% SDS-PAGE and transferred to PVDF (polyvinylidene difluoride, Millipore, USA) membranes. The PVDF membranes were pre-treated with QuickBlock Blocking Buffer (P0235, Beyotime) prior to incubation with the respective primary antibodies: rabbit anti-Alix (12,422–1-AP; Proteintech, Wuhan, China), rabbit anti-GM130 (12,480; Proteintech), rabbit anti-TSG101 (14,497–1-AP; Proteintech), anti-ATF6 (#65,880; CST, Beverly, MA, USA), anti-CD63 (AF5117, affinity), anti-ATF4 (ab216839; Abcam, Cambridge, UK), anti-GRP78 (#3177; CST), anti-LAMP1 (ab24170; Abcam), anti-mTORC1 (ab134903; Abcam), anti-TFEB (ab134903; Abcam), anti-PERK (ab229912; Abcam), anti-Alix (#92,880; CST), and anti-IRE1 (ab37073; Abcam) antibodies. The enzyme-tagged secondary antibodies were applied to the PVDF membrane for a duration of one hour at room temperature. Subsequently, the protein bands were illuminated using an advanced chemiluminescence detection kit, captured via an imaging apparatus (Bio-Rad, ChemiDoc MP).

### EVs isolation and identification

EVs were extracted from cell culture supernatants via differential ultracentrifugation [57]. Briefly, the supernatants underwent sequential centrifugation at 300 × g for 15 min, followed by 2,000 × g for 15 min at 4 °C, and finally at 110,000 × g for 70 min using an Optima XPN-100 ultracentrifuge equipped with an SW32Ti rotor (Beckman Coulter, Brea, CA, USA) [34].

The pelleted EVs were then resuspended in PBS and preserved at −80°C for subsequent analysis. The morphology of EVs was examined with a transmission electron microscope (TEM, JEM-1400FLASH), and their size distribution was determined by nanoparticle tracking analysis (NTA, Particle Metrix, Meerbusch, Germany). A BCA protein assay kit was used to measure the protein levels in EVs and EVs yield was ascertained by calculating the ratio of the number of EVs particles to the total cellular protein content. In order to visually display the data, the value of the control group was set to 1, and the data of the experimental group was expressed as the fold change relative to the control group.

Western blotting was used to examine anti-TSG101 and anti-Alix and GM130.

### TCMK-1 cell uptake of EVs

Cellular uptake assays were performed with PKH26 (Sigma, MO, USA)-labeled EVs. Transformed C3H mouse kidney-1 (TCMK-1) cells were incubated with PKH26-labeled EVs (10 μg/mL) at 37 °C for 1 h. The cells were subsequently washed with PBS and stained with DiO (C1038, Beyotime). The cells were observed using CLSM.

### Animal experiments

Male C57BL/6J mice (21–23 g) were purchased from GemPharmatech (Jiangsu, China). The animals were housed in individual cages with a 12-h light–dark cycle and unlimited access to standard food and sterile water. To determine the role of EVs in mice with kidney stones, the mice were divided into 4 groups, including healthy control (CON), glyoxylic acid (GLA), glyoxylic acid plus EVs derived from BMDMs induced by COM (GLA + COM-EVs) and glyoxylic acid plus EVs derived from BMDMs induced by medium (GLA + C-EVs), *n* = 5 mice. The mice were intraperitoneally injected with GLA (80 mg/kg/day; Sigma) for 7 consecutive days. For EVs treatment, the mice were injected with COM-EVs or C-EVs (80 μg/100 μL of PBS/per mouse) via the tail vein on Days 2, 4, and 6 during modeling [58]. On Day 7, the mice were examined, and on Day 8, urine, blood, and kidneys were collected.

AAV9-mediated PERK knockdown in macrophages was performed in vivo. The AAVs were obtained from GeneChem Company (Shanghai, China). The adeno-associated virus vector expressing short hairpin RNA (shRNA) targeting the sequence of PERK gene (TGCTGATGATTACCAAGGACC) and negative control (TTCTCCGAACGTGTCACGT) were synthesized and cloned into GV407 (pAAV-F4/80p-EGFP-shRNA) vector with BsmBI sites (purchased from Shanghai Genechem Co., Ltd.), recombinant vector was detected by DNA sequencing. The mice were slowly injected with 100 μL of the AAV9-packaged PERK knockdown plasmid at a concentration of 2 × 10^11^ vg/mL through the renal vein. The mice were infected for 30 days. The mice were randomly divided into 4 groups (*n* = 5 mice): a control group (Control), a glyoxylate model group (GLA), a negative control group (GLA + NC-AAV), a glyoxylate model group and an AAV group (GLA + AAV-shPERK). Intraperitoneal injection of glyoxylate was performed for 7 days.

### In vivo tracking of EVs

PKH26-labeled EVs (80 μg/100 μL in PBS per mouse) or an equivalent volume of PKH26 alone were administered intravenously via the tail vein to C57BL/6J mice. At 4 h after injection, the heart, liver, spleen, lungs, and kidneys were collected and observed using an IVIS spectral optical imaging system (Perkin Elmer, Waltham, MA, USA).

### Frozen immunohistochemistry

Frozen kidney were sliced to 7 mm thick sections in a cryostat and fixed with 4% polyformaldehyde for 10 min, left to dry and then washed 3 times with PBS. Sections were blocked with 2% goat serum for 1 h prior to overnight incubation with anti-mouse FITC-conjugated cytokeratin 18 (FITC-66187, proteintech, 1:50 dilution) at 4°C. Following PBS wash, kidney sections were mounted with antifade mounting medium (with DAPI) and a glass coverslip. Images were acquired using the automatic inverted fluorescence microscope (Olympus, IX83, Japan).

### Immunofluorescence staining

The cells were incubated with mouse anti-TFEB (1:50; Proteintech), DiO and DAPI (C1005; Beyotime) antibodies. Fluorescence was measured by confocal microscopy (Nikon). The ROS levels in the cells were analyzed using DCFH-DA (R252, Dojindo).

### Flow cytometry of BMDM cells

After the cells were digested down with trypsin, 3 volumes of complete medium were added and collected by centrifugation. The cell suspension were resuspended in staining buffer and incubated with the following surface antibodies: anti-CD45 (561088, BD Cytofix™), and anti-F4/80 (569223, BD Cytofix™), anti-TLR4 (145405, BioLegend). Incubation was performed for 30 min at RT in the dark. Cells were treated with Fixable Viability Stain 780 (565388, BD Cytofix™) for 15 min at RT. Cells were fixed and permeabilizated (BD Cytofix™). Intracellular staining for anti-iNOS (56-5920-82, ThermoFiusher) was performed by incubating with a specific antibody for 30 min at RT. Cells were washed with PBS and resuspended in flow cytometry staining buffer (PBS with 1% BSA). Data were acquired using a flow cytometer (Cytek, Aurora CS, USA) and analyzed with software (FlowJo v10.8).

### Flow cytometry of kidney

Kidney were mechanically dissociated using a sterile scalpel and gently homogenized by pressing through a 70 μm filter. The cell suspension was mixed with 4 volumes of red blood cell lysis buffer and incubated for 3 min at RT. The reaction was quenched by adding excess PBS, followed by centrifugation at 500 g for 5 min to lyse red blood cells. The pellet was resuspended in 500 μL PBS. Subsequently, cells were resuspended in staining buffer and incubated with the following surface antibodies: anti-CD45, anti-F4/80, and anti-TLR4. Incubation was performed for 30 min at RT in the dark. Cells were treated with Fixable Viability Stain 780 for 15 min at RT. Cells were fixed and permeabilizated). Intracellular staining for anti-iNOS was performed by incubating with a specific antibody for 30 min at RT. Cells were washed with PBS and resuspended in flow cytometry staining buffer (PBS with 1% BSA).

Intracellular staining for anti-PERK (82,534–1-RR) was performed by incubating with a specific antibody for 30 min at RT. Cells were washed with PBS and resuspended in flow cytometry staining buffer. Rabbit anti-Mouse IgG (H + L) Cross-Adsorbed Secondary Antibody, Alexa Fluor 594 Dye (A-11062, ThermoFiusher, 1:1000 dilution) was performed by incubating with a specific antibody for 30 min at RT. Cells were washed with PBS and resuspended in flow cytometry staining buffer.

Data were acquired using a flow cytometer and analyzed with software.

### Proteomic analysis of EVs

Total protein extraction and peptide preparation were performed using the Majorbio microprotein kit. Samples were homogenized in Extract reagent (Reagent 1) followed by 10 min low-temperature non-contact sonication and centrifugation (14,000 rpm, 10 min). The supernatant was subjected to sequential treatment with Dilute (Reagent 2), reduction using Reagent 3 (55°C, 30 min), alkylation with Reagent 4 (RT, 30 min), tryptic digestion with Reagent 5 (37°C, 16 h), and acidification using Reagent 6 (45°C, 30 min). Desalted peptides (C18 Tip) were vacuum-concentrated and reconstituted in 0.1% formic acid for UV quantification (NanoDrop One). LC–MS/MS analysis was conducted on a VanquishNeo-Orbitrap Astral system using a uPAC column (75 μm × 5.5 cm) with 2%−80% ACN gradient (180 SPD). DIA acquisition covered m/z 380–980 (MS1) and 150–2000 (MS2). Proteins were identified via Spectronaut 19 (Protein/Peptide FDR ≤ 0.01, XIC width ≤ 75 ppm) requiring ≥ 1 unique peptide. Differential expression thresholds were set at |FC|> 1.2 and *p* < 0.05 (Student's t-test). Functional enrichment (GO/KEGG) and PPI networks (STRING v11.5) were analyzed through Majorbio Cloud platform.

### mRNA sequencing analysis of kidney

Total RNA was extracted from the tissue using TRIzol® Reagent according the manufacturer’s instructions. Then RNA quality was determined by 5300 Bioanalyser (Agilent) and quantified using the ND-2000 (NanoDrop Technologies, USA) were used to construct a sequencing library. The RNA sequencing transcriptome library was prepared following the TruSeqTM RNA Sample Preparation Kit from Illumina (San Diego, CA). cDNA was synthesized using a SuperScript double-stranded cDNA synthesis kit (Invitrogen) with random hexamer primers (Illumina). Libraries were size selected for cDNA target fragments of 300 bp on 2% Low Range Ultra Agarose followed by PCR amplification using Phusion DNA polymerase (New England Biolabs, Ipswich, MA, USA). The expression of each transcript was calculated according to the tran- scripts per million reads method.

### Data presentation and statistical analysis

The data are shown as the mean ± SEM and were analyzed using GraphPad Prism software (version 8.0). Significance is determined at *p* < 0.05. The normality and variance of the data were assessed before comparison. All normally distributed data were analyzed using two-tailed Student’s t tests or one-way ANOVA for multiple groups.

## Supplementary Information

Supplementary Material 1.

## Data Availability

All author declared no conflict of interest.

## References

[1] Hill AJ, Basourakos SP, Lewicki P, Wu X, Arenas-Gallo C, Chuang D, et al. Incidence of kidney stones in the United States: the continuous national health and nutrition examination survey. J Urol. 2022;207:851–6. 10.1097/ju.0000000000002331. 34854755 10.1097/JU.0000000000002331

[2] Bargagli M, Scoglio M, Howles SA, Fuster DG. Kidney stone disease: risk factors, pathophysiology and management. Nat Rev Nephrol. 2025;21:794–808. 10.1038/s41581-025-00990-x. 40790363 10.1038/s41581-025-00990-x

[3] Singh P, Harris PC, Sas DJ, Lieske JC. The genetics of kidney stone disease and nephrocalcinosis. Nat Rev Nephrol. 2022;18:224–40. 10.1038/s41581-021-00513-4. 34907378 10.1038/s41581-021-00513-4

[4] Franklin BS, Mangan MS, Latz E. Crystal formation in inflammation. Annu Rev Immunol. 2016;34:173–202. 10.1146/annurev-immunol-041015-055539. 26772211 10.1146/annurev-immunol-041015-055539

[5] Newton K, Strasser A, Kayagaki N, Dixit VM. Cell death. Cell. 2024;187:235–56. 10.1016/j.cell.2023.11.044. 38242081 10.1016/j.cell.2023.11.044

[6] Duan C, Li B, Liu H, Zhang Y, Yao X, Liu K, et al. Sirtuin1 Suppresses Calcium Oxalate Nephropathy via Inhibition of Renal Proximal Tubular Cell Ferroptosis Through PGC‐1α‐mediated Transcriptional Coactivation. Adv Sci (Weinh). 2024;11:e2408945. 10.1002/advs.202408945. 39498889 10.1002/advs.202408945 PMC11672264

[7] Aggarwal KP, Narula S, Kakkar M, Tandon C. Nephrolithiasis: molecular mechanism of renal stone formation and the critical role played by modulators. Biomed Res Int. 2013;2013:292953. 10.1155/2013/292953. 24151593 10.1155/2013/292953 PMC3787572

[8] Fasano JM, Khan SR. Intratubular crystallization of calcium oxalate in the presence of membrane vesicles: an in vitro study. Kidney Int. 2001;59:169–78. 10.1046/j.1523-1755.2001.00477.x. 11135069 10.1046/j.1523-1755.2001.00477.x

[9] An L, Wu W, Li S, Lai Y, Chen D, He Z, et al. *Escherichia coli* aggravates calcium oxalate stone formation via PPK1/flagellin-mediated renal oxidative injury and inflammation. Oxid Med Cell Longev. 2021;2021:9949697. 10.1155/2021/9949697. 34336124 10.1155/2021/9949697 PMC8292073

[10] Canela VH, Bowen WS, Ferreira RM, Syed F, Lingeman JE, Sabo AR, et al. A spatially anchored transcriptomic atlas of the human kidney papilla identifies significant immune injury in patients with stone disease. Nat Commun. 2023;14:4140. 10.1038/s41467-023-38975-8. 37468493 10.1038/s41467-023-38975-8 PMC10356953

[11] Sun C, Xie G, Huang F, Liu X. Effects of calcium oxalate on expression of clusterin and lower urinary tract symptoms in prostatitis and benign prostatic hyperplasia patients with calculi. Med Sci Monit. 2018;24:9196–203. 10.12659/MSM.911505. 30560838 10.12659/MSM.911505 PMC6320640

[12] Khan SR, Rodriguez DE, Gower LB, Monga M. Association of Randall plaque with collagen fibers and membrane vesicles. J Urol. 2012;187:1094–100. 10.1016/j.juro.2011.10.125. 22266007 10.1016/j.juro.2011.10.125 PMC3625933

[13] Duffield JS, Lupher M, Thannickal VJ, Wynn TA. Host responses in tissue repair and fibrosis. Annu Rev Pathol. 2013;8:241–76. 10.1146/annurev-pathol-020712-163930. 23092186 10.1146/annurev-pathol-020712-163930 PMC3789589

[14] Locati M, Curtale G, Mantovani A. Diversity, mechanisms, and significance of macrophage plasticity. Annu Rev Pathol. 2020;15:123–47. 10.1146/annurev-pathmechdis-012418-012718. 31530089 10.1146/annurev-pathmechdis-012418-012718 PMC7176483

[15] Mass E, Nimmerjahn F, Kierdorf K, Schlitzer A. Tissue-specific macrophages: how they develop and choreograph tissue biology. Nat Rev Immunol. 2023;23:563–79. 10.1038/s41577-023-00848-y. 36922638 10.1038/s41577-023-00848-y PMC10017071

[16] Chen C, Wang J, Guo Y, Li M, Yang K, Liu Y, et al. Monosodium Urate Crystal-Induced Pyroptotic Cell Death in Neutrophil and Macrophage Facilitates the Pathological Progress of Gout. Small. 2023:e2308749. 10.1002/smll.202308749 10.1002/smll.202308749 38161265

[17] Tatano Y, Shimizu T, Sano C, Tomioka H. Roles of autophagy in killing of mycobacterial pathogens by host macrophages - effects of some medicinal plants. Eur J Microbiol Immunol (Bp). 2024. 10.1556/1886.2023.00062. 38349363 10.1556/1886.2023.00062 PMC10895364

[18] De Meyer GRY, Zurek M, Puylaert P, Martinet W. Programmed death of macrophages in atherosclerosis: mechanisms and therapeutic targets. Nat Rev Cardiol. 2024. 10.1038/s41569-023-00957-0. 38163815 10.1038/s41569-023-00957-0

[19] Wang S, Wang J, Chen Z, Luo J, Guo W, Sun L, et al. Targeting M2-like tumor-associated macrophages is a potential therapeutic approach to overcome antitumor drug resistance. NPJ Precis Oncol. 2024;8:31. 10.1038/s41698-024-00522-z. 38341519 10.1038/s41698-024-00522-z PMC10858952

[20] Taguchi K, Okada A, Unno R, Hamamoto S, Yasui T. Macrophage function in calcium oxalate kidney stone formation: a systematic review of literature. Front Immunol. 2021;12:673690. 10.3389/fimmu.2021.673690. 34108970 10.3389/fimmu.2021.673690 PMC8182056

[21] Singh AK, Haque M, O’Sullivan K, Chourasia M, Ouseph MM, Ahmed S. Suppression of monosodium urate crystal-induced inflammation by inhibiting TGF-beta-activated kinase 1-dependent signaling: role of the ubiquitin proteasome system. Cell Mol Immunol. 2021;18:162–70. 10.1038/s41423-019-0284-3. 31511642 10.1038/s41423-019-0284-3 PMC7853128

[22] He J, Cao Y, Zhu Q, Wang X, Cheng G, Wang Q, et al. Renal macrophages monitor and remove particles from urine to prevent tubule obstruction. Immunity. 2024;57(106–23):e7. 10.1016/j.immuni.2023.12.003. 10.1016/j.immuni.2023.12.003 38159573

[23] Nakayama M. Macrophage recognition of crystals and nanoparticles. Front Immunol. 2018;9:103. 10.3389/fimmu.2018.00103. 29434606 10.3389/fimmu.2018.00103 PMC5796913

[24] Chiangjong W, Thongboonkerd V. Calcium oxalate crystals increased enolase-1 secretion from renal tubular cells that subsequently enhanced crystal and monocyte invasion through renal interstitium. Sci Rep. 2016;6:24064. 10.1038/srep24064. 27045290 10.1038/srep24064 PMC4820722

[25] Pan Z, Sun W, Chen Y, Tang H, Lin W, Chen J, et al. Extracellular vesicles in tissue engineering: biology and engineered strategy. Adv Healthc Mater. 2022;11:e2201384. 10.1002/adhm.202201384. 36053562 10.1002/adhm.202201384

[26] Bruno S, Kholia S, Deregibus MC, Camussi G. The Role of Extracellular Vesicles as Paracrine Effectors in Stem Cell-Based Therapies. Adv Exp Med Biol. 2019;1201:175–93. 10.1007/978-3-030-31206-0_9. 31898787 10.1007/978-3-030-31206-0_9

[27] Wang Y, Zhao M, Liu S, Guo J, Lu Y, Cheng J, et al. Macrophage-derived extracellular vesicles: diverse mediators of pathology and therapeutics in multiple diseases. Cell Death Dis. 2020;11:924. 10.1038/s41419-020-03127-z. 33116121 10.1038/s41419-020-03127-z PMC7595091

[28] Zhang J, Kumar S, Jayachandran M, Herrera Hernandez LP, Wang S, Wilson EM, et al. Excretion of urine extracellular vesicles bearing markers of activated immune cells and calcium/phosphorus physiology differ between calcium kidney stone formers and non-stone formers. BMC Nephrol. 2021;22:204. 10.1186/s12882-021-02417-8. 34074247 10.1186/s12882-021-02417-8 PMC8170929

[29] Khan SR, Pearle MS, Robertson WG, Gambaro G, Canales BK, Doizi S, et al. Kidney stones. Nat Rev Dis Primers. 2016;2:16008. 10.1038/nrdp.2016.8. 27188687 10.1038/nrdp.2016.8 PMC5685519

[30] Mulay SR, Anders HJ. Crystal nephropathies: mechanisms of crystal-induced kidney injury. Nat Rev Nephrol. 2017;13:226–40. 10.1038/nrneph.2017.10. 28218266 10.1038/nrneph.2017.10

[31] Kumar P, Saini K, Saini V, Mitchell T. Oxalate alters cellular bioenergetics, redox homeostasis, antibacterial response, and immune response in macrophages. Front Immunol. 2021;12:694865. 10.3389/fimmu.2021.694865. 34745086 10.3389/fimmu.2021.694865 PMC8566947

[32] Sun X, Meng H, Wan W, Xie M, Wen C. Application potential of stem/progenitor cell-derived extracellular vesicles in renal diseases. Stem Cell Res Ther. 2019;10:8. 10.1186/s13287-018-1097-5. 30616603 10.1186/s13287-018-1097-5 PMC6323814

[33] Abels ER, Breakefield XO. Introduction to extracellular vesicles: biogenesis, RNA cargo selection, content, release, and uptake. Cell Mol Neurobiol. 2016;36:301–12. 10.1007/s10571-016-0366-z. 27053351 10.1007/s10571-016-0366-z PMC5546313

[34] Wang Y, Liu S, Li L, Li L, Zhou X, Wan M, et al. Peritoneal M2 macrophage-derived extracellular vesicles as natural multitarget nanotherapeutics to attenuate cytokine storms after severe infections. J Control Release. 2022;349:118–32. 10.1016/j.jconrel.2022.06.063. 35792186 10.1016/j.jconrel.2022.06.063 PMC9257240

[35] Furmanik M, van Gorp R, Whitehead M, Ahmad S, Bordoloi J, Kapustin A, et al. Endoplasmic reticulum stress mediates vascular smooth muscle cell calcification via increased release of Grp78 (glucose-regulated protein, 78 kDa)-loaded extracellular vesicles. Arterioscler Thromb Vasc Biol. 2021;41:898–914. 10.1161/ATVBAHA.120.315506. 33297752 10.1161/ATVBAHA.120.315506 PMC7837691

[36] Tian K, Wei J, Wang R, Wei M, Hou F, Wu L. Sophoridine derivative 6j inhibits liver cancer cell proliferation via ATF3 mediated ferroptosis. Cell Death Discov. 2023;9:296. 10.1038/s41420-023-01597-6. 37580343 10.1038/s41420-023-01597-6 PMC10425377

[37] Shelke GV, Williamson CD, Jarnik M, and Bonifacino JS. Inhibition of endolysosome fusion increases exosome secretion. J Cell Biol. 2023;222. 10.1083/jcb.202209084. 10.1083/jcb.202209084 PMC10202829 37213076

[38] Adams SD, Csere J, D’Angelo G, Carter EP, Romao M, Arnandis T, et al. Centrosome amplification mediates small extracellular vesicle secretion via lysosome disruption. Curr Biol. 2021;31(1403–16):e7. 10.1016/j.cub.2021.01.028. 10.1016/j.cub.2021.01.028 PMC8047808 33592190

[39] Barral DC, Staiano L, Guimas Almeida C, Cutler DF, Eden ER, Futter CE, et al. Current methods to analyze lysosome morphology, positioning, motility and function. Traffic. 2022;23:238–69. 10.1111/tra.12839. 35343629 10.1111/tra.12839 PMC9323414

[40] Rossetto IMU, Santos FR, da Silva HM, Minatel E, Mesquitta M, Salvador MJ, et al. Tempol effect on oxidative and mitochondrial markers in preclinical models for prostate cancer. Toxicol Res. 2024;13:tfae056. 10.1093/toxres/tfae056. 10.1093/toxres/tfae056 PMC11015989 38623092

[41] Wang L, Zhao C, Zheng T, Zhang Y, Liu H, Wang X, et al. Torin 1 alleviates impairment of TFEB-mediated lysosomal biogenesis and autophagy in TGFBI (p. G623_H626del)-linked Thiel-Behnke corneal dystrophy. Autophagy. 2022;18:765–82. 10.1080/15548627.2021.1955469. 34403298 10.1080/15548627.2021.1955469 PMC9037417

[42] Yan L, Chen J, Fang W. Exosomes derived from calcium oxalate-treated macrophages promote apoptosis of HK-2 cells by promoting autophagy. Bioengineered. 2022;13:2442–50. 10.1080/21655979.2021.2012622. 35037827 10.1080/21655979.2021.2012622 PMC8974144

[43] Singhto N, Thongboonkerd V. Exosomes derived from calcium oxalate-exposed macrophages enhance IL-8 production from renal cells, neutrophil migration and crystal invasion through extracellular matrix. J Proteomics. 2018;185:64–76. 10.1016/j.jprot.2018.06.015. 29953960 10.1016/j.jprot.2018.06.015

[44] Yoodee S, Noonin C, Sueksakit K, Kanlaya R, Chaiyarit S, Peerapen P, et al. Effects of secretome derived from macrophages exposed to calcium oxalate crystals on renal fibroblast activation. Commun Biol. 2021;4:959. 10.1038/s42003-021-02479-2. 34381146 10.1038/s42003-021-02479-2 PMC8358035

[45] Arya SB, Collie SP, Parent CA. The ins-and-outs of exosome biogenesis, secretion, and internalization. Trends Cell Biol. 2024;34:90–108. 10.1016/j.tcb.2023.06.006. 37507251 10.1016/j.tcb.2023.06.006 PMC10811273

[46] Dixson AC, Dawson TR, Di Vizio D, Weaver AM. Context-specific regulation of extracellular vesicle biogenesis and cargo selection. Nat Rev Mol Cell Biol. 2023;24:454–76. 10.1038/s41580-023-00576-0. 36765164 10.1038/s41580-023-00576-0 PMC10330318

[47] Matsui T, Osaki F, Hiragi S, Sakamaki Y, Fukuda M. ALIX and ceramide differentially control polarized small extracellular vesicle release from epithelial cells. EMBO Rep. 2021;22:e51475. 10.15252/embr.202051475. 33724661 10.15252/embr.202051475 PMC8097368

[48] Wei D, Zhan W, Gao Y, Huang L, Gong R, Wang W, et al. RAB31 marks and controls an ESCRT-independent exosome pathway. Cell Res. 2021;31:157–77. 10.1038/s41422-020-00409-1. 32958903 10.1038/s41422-020-00409-1 PMC8027411

[49] Solvik TA, Nguyen TA, Tony Lin YH, Marsh T, Huang EJ, Wiita AP, et al. Secretory autophagy maintains proteostasis upon lysosome inhibition. J Cell Biol. 2022;221. 10.1083/jcb.202110151. 10.1083/jcb.202110151 PMC9036093 35446347

[50] Delorme-Axford E, Klionsky DJ. The LC3-conjugation machinery specifies cargo loading and secretion of extracellular vesicles. Autophagy. 2020;16:1169–71. 10.1080/15548627.2020.1760057. 32401566 10.1080/15548627.2020.1760057 PMC7469661

[51] Koncz A, Turiak L, Nemeth K, Lenzinger D, Barkai T, Lorincz P, et al. Endoplasmin Is a Hypoxia-Inducible Endoplasmic Reticulum-Derived Cargo of Extracellular Vesicles Released by Cardiac Cell Lines. Membranes (Basel). 2023;13. 10.3390/membranes13040431. 10.3390/membranes13040431 PMC10142439 37103858

[52] Papadopoulos C, Meyer H. Detection and clearance of damaged lysosomes by the endo-lysosomal damage response and lysophagy. Curr Biol. 2017;27:R1330–41. 10.1016/j.cub.2017.11.012. 29257971 10.1016/j.cub.2017.11.012

[53] Grange C, Bussolati B. Extracellular vesicles in kidney disease. Nat Rev Nephrol. 2022;18:499–513. 10.1038/s41581-022-00586-9. 35641620 10.1038/s41581-022-00586-9 PMC9152665

[54] Peerapen P, Thongboonkerd V. Protective Cellular Mechanism of Estrogen Against Kidney Stone Formation: A Proteomics Approach and Functional Validation. Proteomics. 2019;19:e1900095. 10.1002/pmic.201900095. 31475403 10.1002/pmic.201900095

[55] Taller A, Grohe B, Rogers KA, Goldberg HA, Hunter GK. Specific adsorption of osteopontin and synthetic polypeptides to calcium oxalate monohydrate crystals. Biophys J. 2007;93:1768–77. 10.1529/biophysj.106.101881. 17496021 10.1529/biophysj.106.101881 PMC1948058

[56] Rubio-Navarro A, Guerrero-Hue M, Martin-Fernandez B, Cortegano I, Olivares-Alvaro E, de Las HN, et al. Phenotypic Characterization of Macrophages from Rat Kidney by Flow Cytometry. J Vis Exp. 2016. 10.3791/54599. 27805599 10.3791/54599 PMC5092211

[57] Zhao M, Liu S, Wang C, Wang Y, Wan M, Liu F, et al. Mesenchymal stem cell-derived extracellular vesicles attenuate mitochondrial damage and inflammation by stabilizing mitochondrial DNA. ACS Nano. 2021;15:1519–38. 10.1021/acsnano.0c08947. 33369392 10.1021/acsnano.0c08947

[58] Okada A, Nomura S, Higashibata Y, Hirose M, Gao B, Yoshimura M, et al. Successful formation of calcium oxalate crystal deposition in mouse kidney by intraabdominal glyoxylate injection. Urol Res. 2007;35:89–99. 10.1007/s00240-007-0082-8. 17393196 10.1007/s00240-007-0082-8

