# Supplementary material for: ER stress induced extracellular vesicles secretion from macrophages promotes calcium oxalate crystals formation in kidney
Source: Mol Biomed. 2025 Nov 17;6:110. doi: 10.1186/s43556-025-00351-x (PMC12623569; doi:10.1186/s43556-025-00351-x)
Supplement: Supplementary file 1 — Supplementary Material 1. [file 43556_2025_351_MOESM1_ESM.pdf]

ER Stress Induced Extracellular Vesicles Secretion from Macrophages Promotes Calcium Oxalate Crystals Formation in Kidney

Yiqiong Yuan[1,2#], Yucheng Ma[1#], Lunzhi Dai[2], Xi Jin[1]*, Shiqian Qi[1,2], Zhaofa Yin[1,3]*

1 Department of Urology, Institute of Urology (Laboratory of Reconstructive Urology), West China Hospital, Sichuan University, Chengdu, Sichuan, P.R. China.

2 State Key Laboratory of Biotherapy, West China Hospital, Sichuan University, and National Collaborative Innovation Center, Chengdu, Sichuan, P.R. China.

3 Department of Urology, Loudi Central Hospital of Hunan Province, Loudi, Hunan, P.R. China.

[#] Yiqiong Yuan and Yucheng Ma contributed equally as first authors.

Correspondence:

Zhaofa Yin, Department of Urology, Loudi Central Hospital of Hunan Province, No. 51 Changqing East Street, Loudi, Hunan, 417000, China. E-mail: yinzhaofa@ldzxyy.com

Xi Jin, Department of Urology, Institute of Urology (Laboratory of Reconstructive Urology), West China Hospital, Sichuan University, No. 37 Guo Xue Xiang, Chengdu, Sichuan, 610041, P.R. China. E-mail: jinxi@wchscu.cn

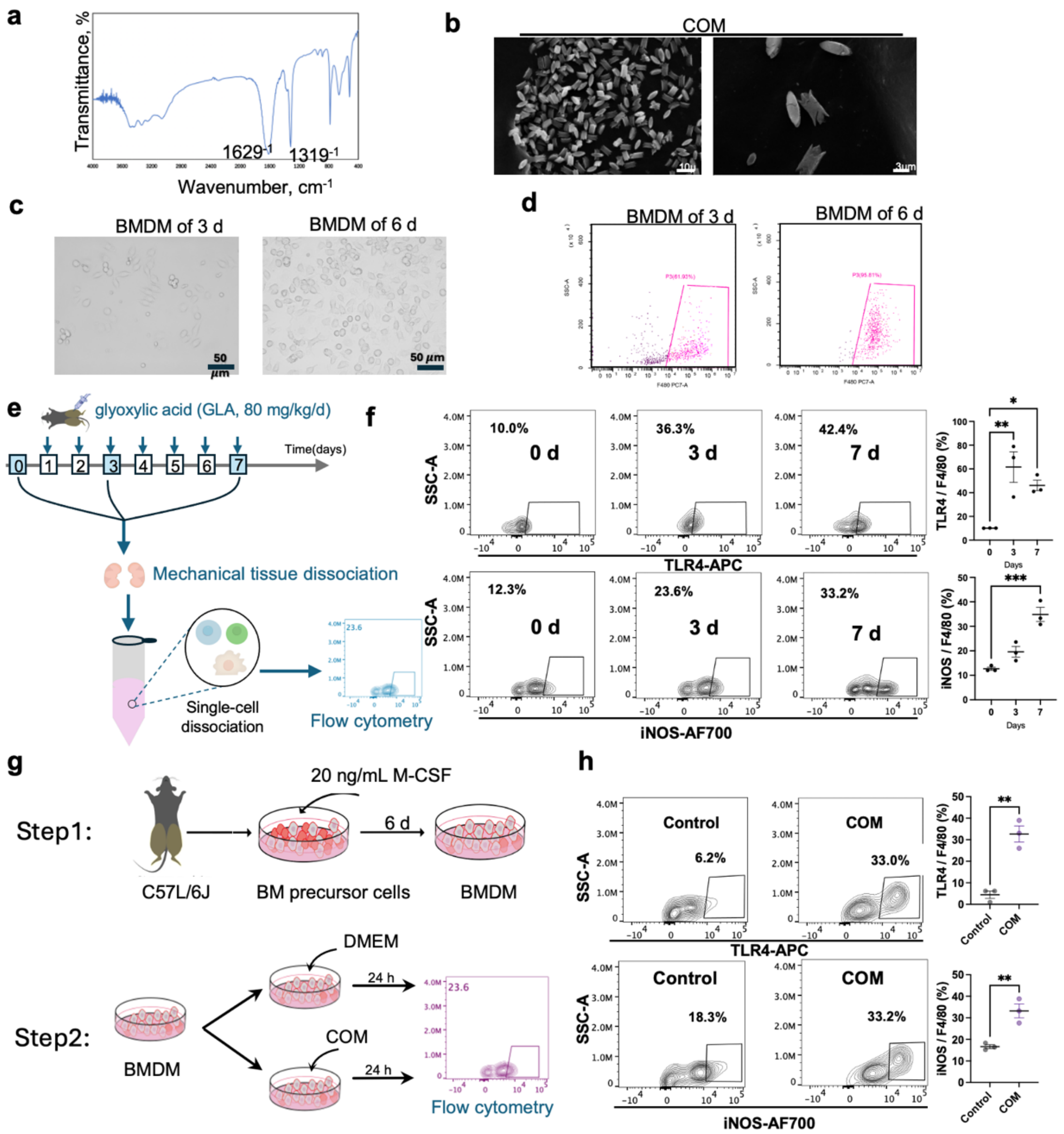

Supplementary data Fig.1: BMDMs have comparable phenotypes to kidney macrophages. a Fourier transform infrared (FT-IR) spectra of COM. **b** SEM image of the COM; magnification, 1000×. **c** BMDMs were imaged via an inverted phase-contrast light microscope. **d**, Flow cytometry analysis of F4/80 in BMDMs. **e** Experimental scheme for phenotypes of kidney macrophages via flow cytometry (FCM). **f** FCM staining analysis of TLR4/iNOS expression in glyoxylic acid treated mouse kidney 0-7 days. **g** Experimental scheme of BMDM isolation and FCM staining analysis of TLR4/iNOS expression in BMDM. **h** FCM staining analysis of TLR4/iNOS expression in BMDM.

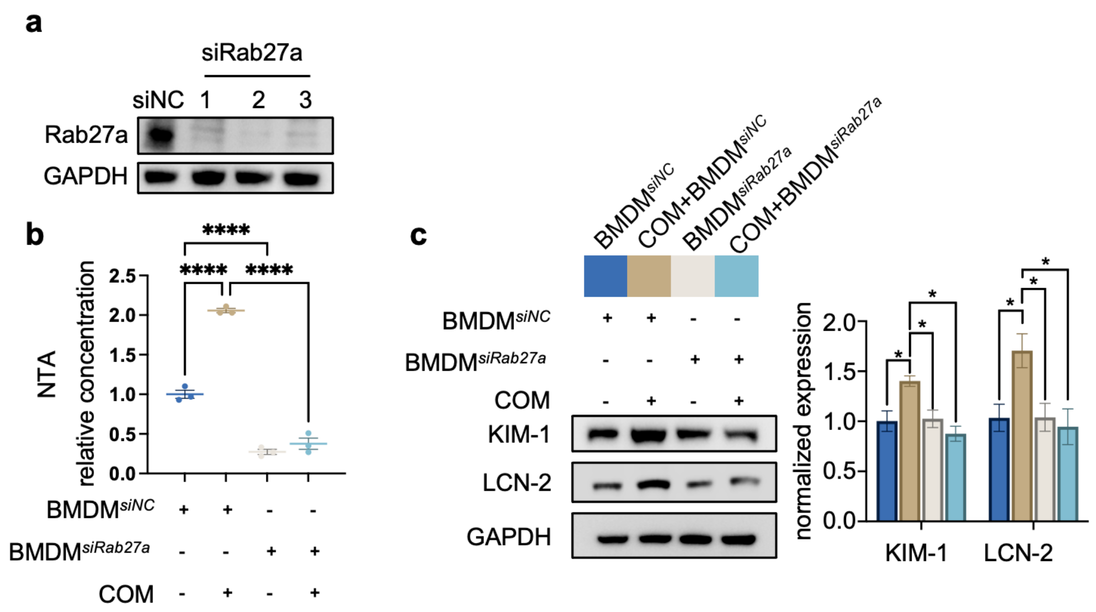

Supplementary data Fig.2 a After transfecting BMDM cells with plasmids for siNC or siRab27a for 48 h, the cells were harvested to analyze the protein expression levels of Rab27a by Western blotting. **b** NTA analysis of the concentration of EVs production by BMDM under the four conditions: with and without CaOx stimulation, and with and without Rab27a knockdown. **c** Rab27a knockdown BMDM stimulated with CaOx crystals (COM) were placed in the upper chamber, while TCMK-1 cells were in the lower chamber. TCMK-1 cells were harvested to analyze the protein expression levels of KIM-1 and LCN-2 by Western blotting. Relative quantification (**left**) of the data presented in (**right**). All data are presented as means ± SEM. Significance was determined by one-way ANOVA with Tukey's multiple comparisons test, n=3, * means $p < 0.05$, **** means $p < 0.0001$.

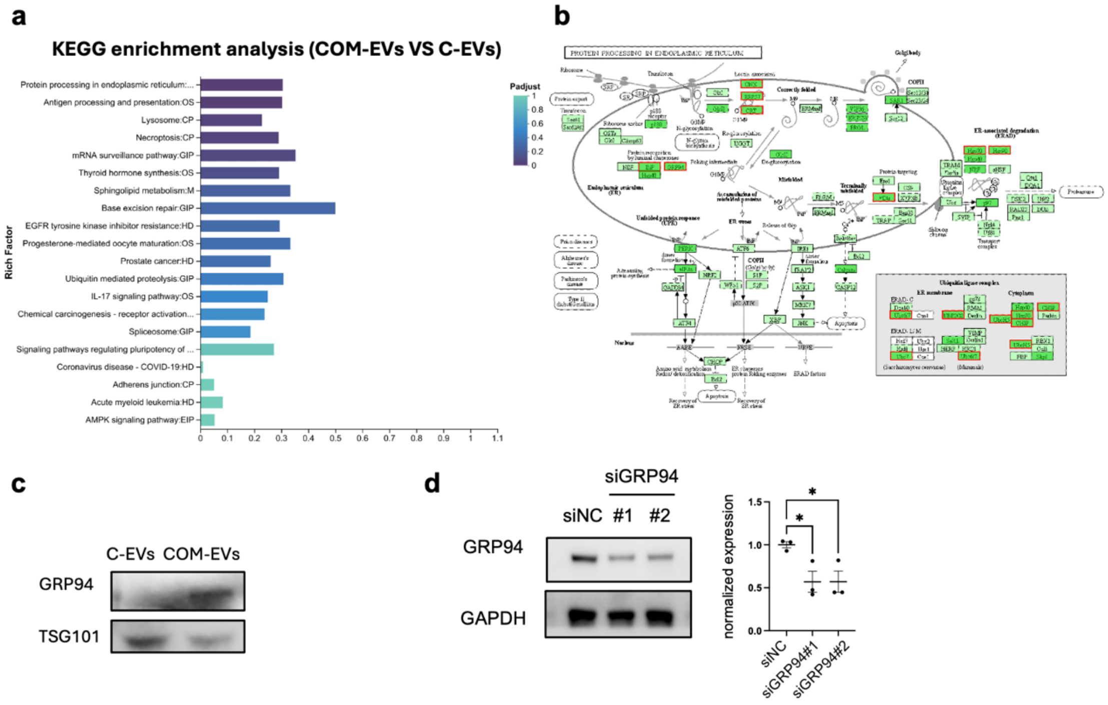

Supplementary data Fig.3 a KEGG enrichment map of protein in COM-EVs and C-EVs. **b** Protein processing in ER. Red labeled the differentially expressed protein in COM-EVs and C-EVs. **c** Western blot analysis of the GRP94 in EVs. **d** Western blot analysis of the GRP94 in BMDMs transfected with scrambled siRNA (siNC) or PERK siRNA (siGRP94). Relative quantification (**left**) of the data presented in (**right**). The data are presented as means ± SEM. Significance was determined by one-way ANOVA with Tukey's multiple comparisons test, n = 3 biologically independent samples, $*p < 0.05$.

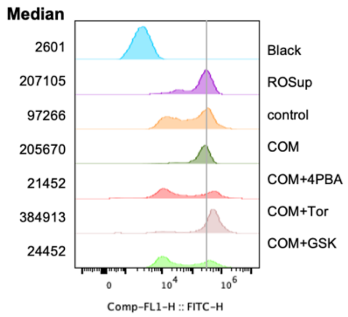

Supplementary data Fig.4 The levels of ROS in BMDMs treated with COM in the absence or presence of 4PBA or Tor for 24 h. Untreated BMDMs served as a negative control. BMDMs treated with ROSup were used as positive controls. Flow cytometry analysis of the level of ROS.

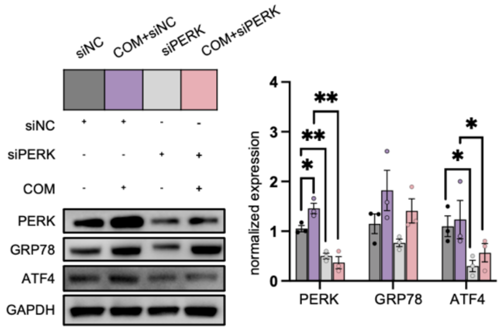

Supplementary data Fig.5 The expression of PERK and activation of the downstream ATF4 pathway were significantly reduced in COM-stimulated BMDMs after treatment with siPERK (COM+siPERK). Western blot analysis of the PERK pathway-related protein levels of GRP78, PERK, and ATF4 in BMDMs transfected with scrambled siRNA (siNC) or PERK siRNA (siPERK) and then treated with COM for 24 h. Untreated BMDMs served as controls. Relative quantification (**left**) of the data presented in (**right**).

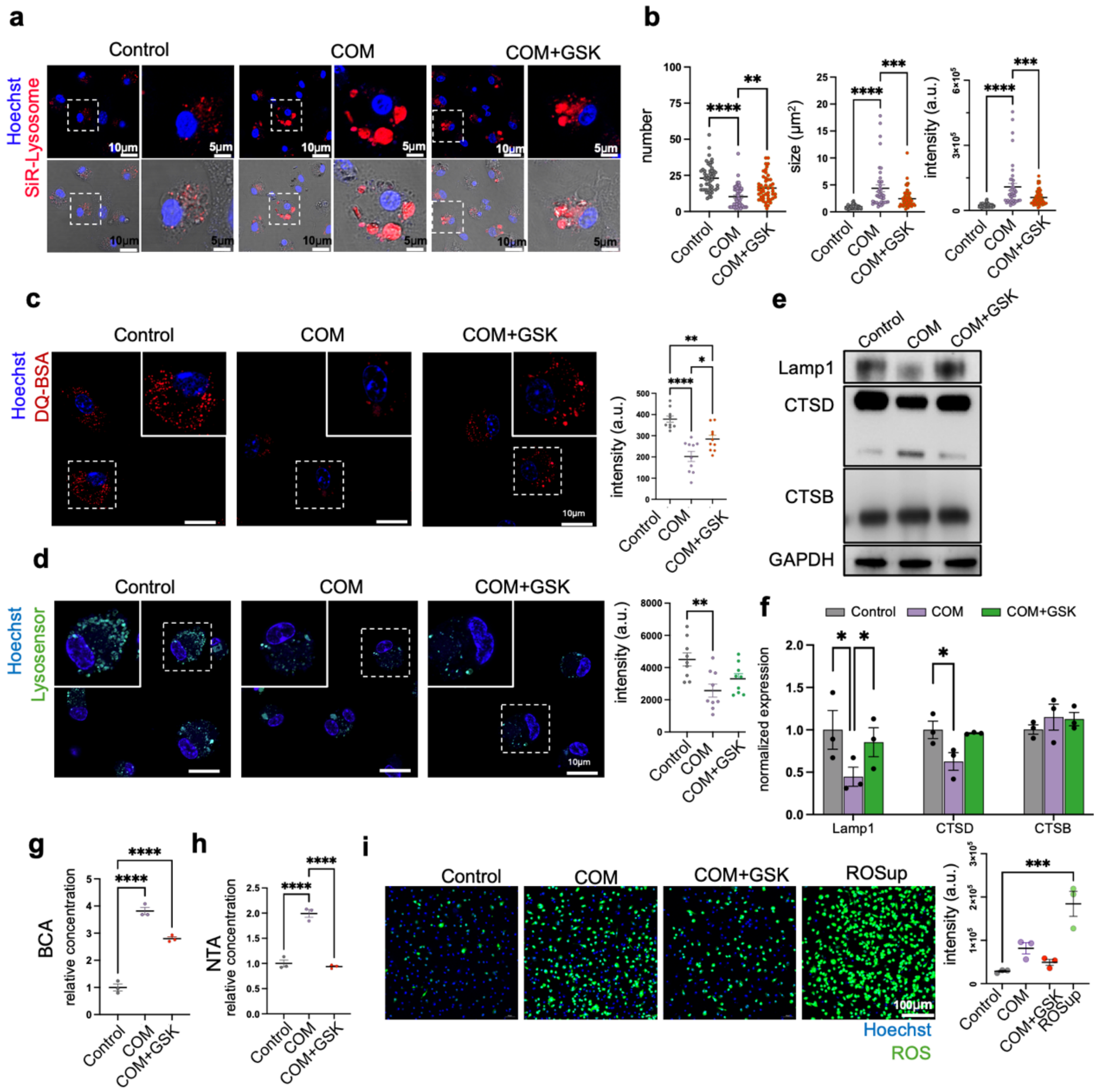

Supplementary data Fig.6: PERK-mediated lysosomal dysfunction induces EVs release during COM stimulation. a-b To detect lysosome function and EVs release, BMDMs were treated with COM in the absence or presence of GSK for 24 h. Untreated BMDMs served as controls. Representative fluorescence images of SiR-Lysosome (Red) staining detected via laser scanning confocal microscopy (**a**) and quantification of the number, size, and fluorescence intensity of red (**b**). **c** Representative fluorescence images of DA-BSA (Red) staining detected via laser scanning confocal microscopy. Relative quantification (**right**) of the data presented in (**left**). **d** Representative fluorescence images of Lysosensor (green) staining detected via laser scanning confocal microscopy. Relative quantification (**right**) of the data presented in (**left**). **e-f** Western blot analysis of the lysosome-related protein levels of Lamp1, Cathepsin D, Cathepsin B in BMDMs treated with COM in the absence or presence of GSK for 24 h. Relative quantification (**f**) of the data presented in (**e**). **g-h** BCA and NTA analysis of the concentration of EVs. **i** Representative fluorescence images and quantitative analysis of the level of ROS**.** The levels of ROS in BMDMs treated with COM in the absence or presence of GSK for 24 h. Untreated BMDMs served as controls. BMDMs treated with ROSup were used as positive controls. Representative fluorescence images of BMDMs treated with or without COM for 24 h. Relative quantification (**right**) of the data presented in (**left**). All the data are presented as means ± SEM. Significance was determined

by one-way ANOVA with Tukey's multiple comparisons test, n = 3 biologically independent samples, ****$p < 0.0001$, ***$p < 0.001$, **$p < 0.01$.

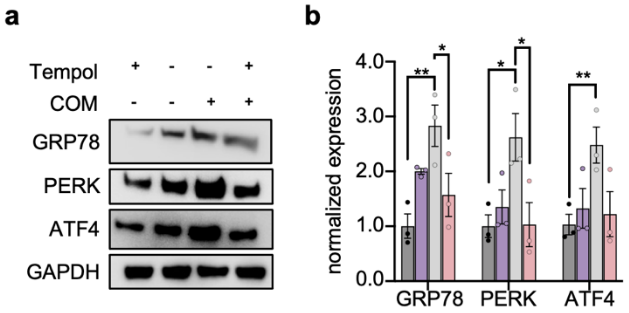

Supplementary data Fig.7 a BMDMs were stimulated with COM crystals for 24 h in the presence or absence of Tempol, and the cells were harvested to analyze the expression levels of GRP78, PERK, and ATF4 by Western blotting. **b** Statistical plot of relative protein expression of GRP78, PERK, and ATF4. All data are presented as means ± SEM. Significance was determined by one-way ANOVA with Tukey's multiple comparisons test, n=3, * means $p < 0.05$, ** means $p < 0.01$.

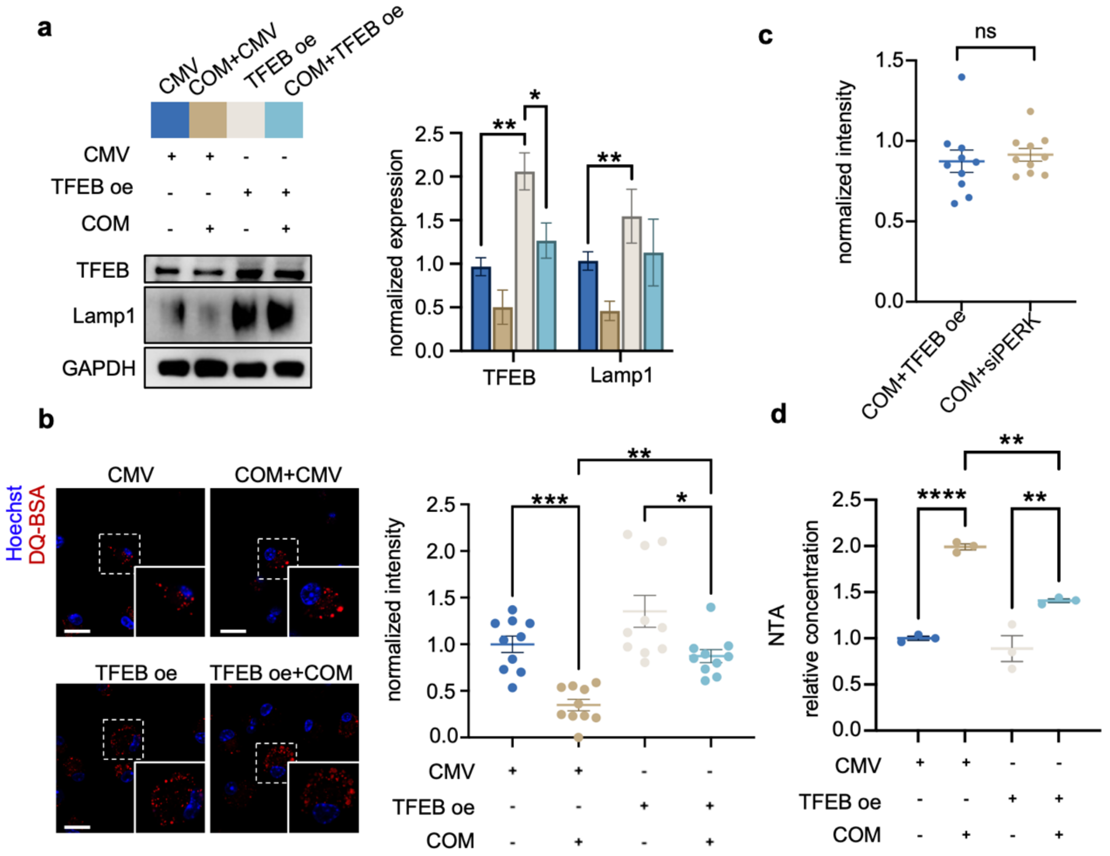

Supplementary data Fig.8 a After transfecting BMDM cells with plasmids for CMV or TFEB overexpression for 48 hours, the cells were harvested to analyze the protein expression levels of TFEB and Lamp1. Relative quantification (**right**) of the data presented in (**left**). **b** 48 h after transfecting BMDM cells with plasmids for CMV or TFEB overexpression, the cells were stained with DQ-BSA and observed using a confocal microscope. Relative quantification (**right**) of the data presented in (**left**). **c** The DQ-BSA fluorescence intensity for both TFEB overexpression and PERK knockdown was normalized against the control group (set to 1). Data are expressed as fold changes relative to the control group (set to 1). **d** The concentrations of EVs released by BMDMs under the four conditions: with and without COM stimulation, and with and without TFEB overexpression were evaluated by NTA. EVs particle and protein concentrations were normalized to the total protein content of cell. Data from the control group were set to 1, and experimental groups are expressed as fold changes relative to the control. All data are presented as means ± SEM. Significance was determined by one-way ANOVA with Tukey's multiple comparisons test, n=3, * means $p < 0.05$, ** means $p < 0.01$, *** means $p < 0.001$, **** means $p < 0.0001$.

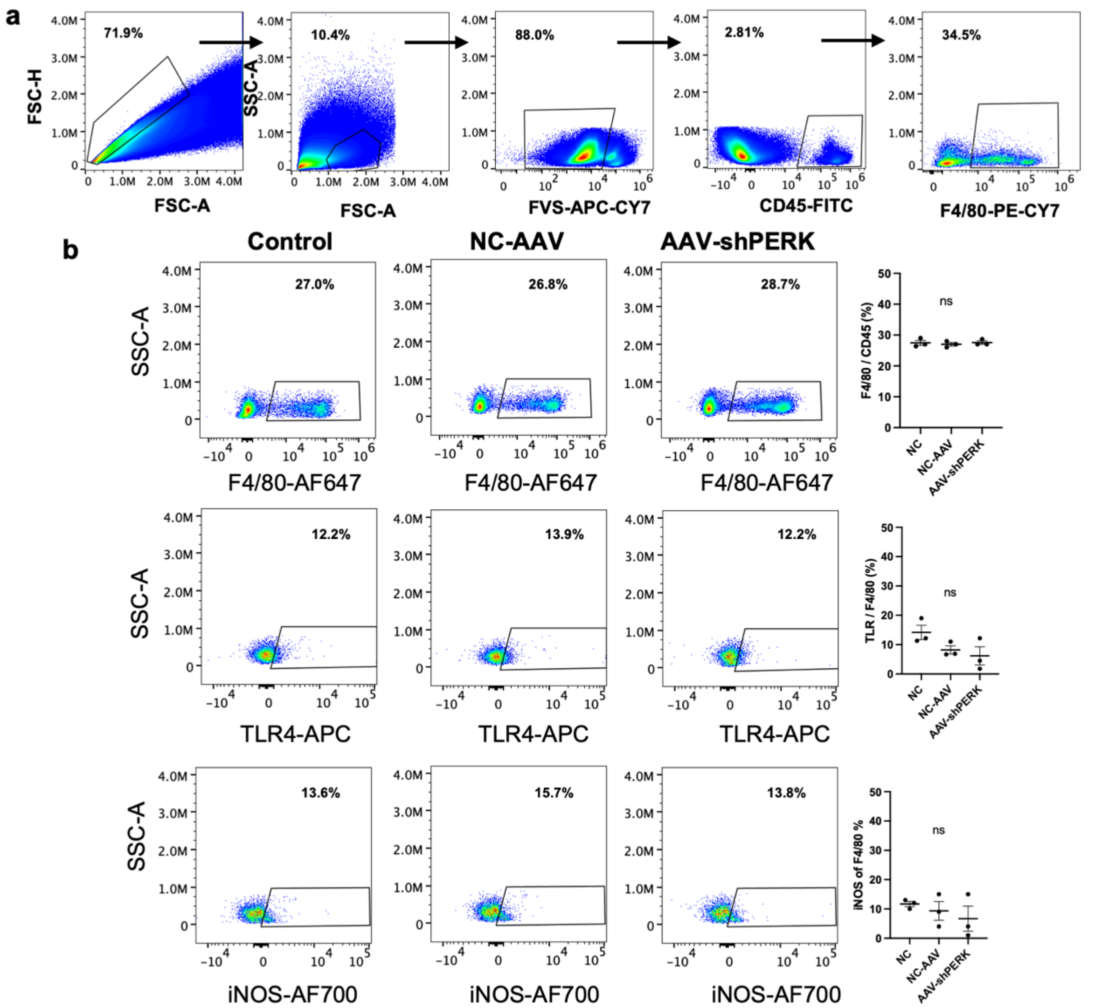

Supplementary data Fig.9 AAV-shPERK could not affect renal macrophage population and activation. a Representative gating strategy for bulk macrophage analyses in kidney. FVS^- cells for live cells; $CD45^+$ for leukocytes; $F4/80^+$ for macrophages. **b** Representative flow cytometry staining analysis of F4/80, TLR4 and iNOS expression in BMDM. Relative quantification (**right**) of the data presented in (**left**). All the data are presented as means ± SEM. Significance was determined by one-way ANOVA with Tukey's multiple comparisons test, n = 3. ns means not significant.

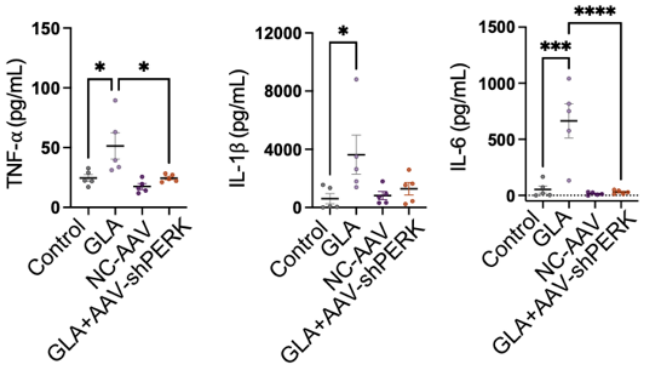

Supplementary data Fig.10 ELISA analysis of the levels of the cytokines TNF-α, IL-6 and IL-1β in the serum of the mice. All the data are presented as means ± SEM. Significance was determined by one-way ANOVA with Tukey's multiple comparisons test, n = 3. ns means not significant, ****$p < 0.0001$, ***$p < 0.001$, *$p < 0.05$.
